# Sex-Related Differences in Myocardial Deformation and Systolic Function in Healthy Individuals: A Systematic Review and Meta-Analysis of Global Longitudinal Strain and Left Ventricular Ejection Fraction

**DOI:** 10.3390/jcm15082859

**Published:** 2026-04-09

**Authors:** Andrea Sonaglioni, Giulio Francesco Gramaglia, Gian Luigi Nicolosi, Massimo Baravelli, Michele Lombardo

**Affiliations:** 1 Division of Cardiology, IRCCS MultiMedica, 20123 Milan, Italy; massimo.baravelli@multimedica.it (M.B.); michele.lombardo@multimedica.it (M.L.); 2 Department of Emergency, Fondazione IRCSS Ca’ Granda, Ospedale Maggiore Policlinico, 20122 Milan, Italy; giulio.gramaglia@unimi.it; 3 Division of Cardiology, Policlinico San Giorgio, 33170 Pordenone, Italy; gianluigi.nicolosi@gmail.com

**Keywords:** global longitudinal strain, speckle-tracking echocardiography, sex differences, myocardial deformation, reference values, meta-analysis

## Abstract

**Background:** Left ventricular global longitudinal strain (GLS) measured by speckle-tracking echocardiography (STE) has become a key marker of myocardial systolic function, yet normal reference values remain heterogeneous, and the magnitude of physiological sex differences is not fully defined. We performed a systematic review and meta-analysis to establish pooled GLS reference estimates in healthy individuals, quantify sex-related differences, and contextualize deformation findings relative to conventional systolic function. **Methods:** A systematic search of PubMed, Scopus, and EMBASE identified observational studies reporting GLS in healthy adults assessed by two-dimensional or three-dimensional STE. Random-effects meta-analysis using standardized mean differences (SMD) compared GLS between women and men. Descriptive pooled reference values were derived using weighted median and interquartile range (IQR) reconstruction from study-level distributions. Meta-regression analyses explored demographic, clinical, and methodological sources of heterogeneity. A complementary analysis evaluated sex-related differences in left ventricular ejection fraction (LVEF) within the same populations. **Results:** Thirty-two studies, including 19,157 healthy individuals, were analyzed. The pooled population had a weighted median age of 47.5 years and 53% female participants. Overall, GLS demonstrated a weighted median of 20.3% (IQR 17.8–22.5). Women showed higher GLS values than men (20.8% [18.4–23.1] vs. 19.4% [17.0–21.6]). Meta-analysis of 28 studies confirmed significantly greater GLS in females (SMD 0.487, 95% CI 0.409–0.565; *p* < 0.001), with consistent findings across imaging modalities and no subgroup interaction. Between-study heterogeneity was substantial (I^2^ = 82.7%), although effect direction was uniform. Meta-regression analyses identified no significant moderators, and sensitivity analyses confirmed stable estimates without publication bias. Segmental analysis demonstrated a physiological base-to-apex strain gradient. In contrast, LVEF was largely comparable between sexes, with no clinically meaningful difference (SMD 0.257, 95% CI 0.186–0.327; *p* < 0.001), indicating preserved global systolic performance despite differences in myocardial deformation. **Conclusions:** GLS demonstrates a consistent physiological range in healthy populations, with women exhibiting higher longitudinal deformation than men, independent of the imaging modality. These findings support the adoption of sex-specific GLS reference values and highlight the complementary roles of deformation and volumetric indices in improving the interpretation of myocardial function and reducing misclassification in clinical practice.

## 1. Introduction

Left ventricular (LV) global longitudinal strain (GLS), derived from speckle-tracking echocardiography (STE), has emerged as a sensitive and reproducible marker of myocardial systolic function, capable of detecting subtle abnormalities in myocardial mechanics beyond conventional indices such as left ventricular ejection fraction (LVEF) [1,2,3,4]. While LVEF remains the cornerstone measure of global systolic performance in clinical practice, deformation imaging provides complementary information by directly quantifying myocardial fiber shortening rather than volumetric chamber emptying. Over the past decade, GLS has progressively transitioned from a research tool to a clinically relevant parameter incorporated into routine echocardiographic assessment and guideline recommendations for the evaluation of cardiomyopathies, cardio-oncology surveillance, and early myocardial dysfunction [5,6].

Despite its widespread adoption, defining normal reference values for GLS remains challenging [7]. Studies conducted in healthy populations using both two-dimensional (2D) and three-dimensional (3D) STE have reported considerable variability in GLS estimates [8,9]. This heterogeneity is partly attributable to differences in vendor technology, image acquisition protocols, post-processing algorithms, and population characteristics such as age, body size, and ethnicity [10,11]. Large population-based cohorts and multicenter investigations have further shown that biological determinants significantly influence myocardial deformation measurements, emphasizing the need for refined normative frameworks and for interpretation of strain parameters alongside established volumetric indices such as LVEF [12,13,14].

Among these determinants, biological sex appears to be one of the most relevant factors influencing myocardial mechanics [15]. Several investigations have reported higher absolute GLS values in women than in men, even in the absence of structural heart disease, suggesting intrinsic sex-related differences in ventricular geometry, myocardial architecture, and contractile mechanics [16]. Potential explanations include smaller ventricular dimensions, differences in loading conditions, hormonal influences, and variations in myocardial contractile reserve [17,18,19]. In contrast, sex-related differences in LVEF appear smaller and less consistent across healthy populations, raising the possibility that myocardial deformation indices capture physiological variability not fully reflected by conventional volumetric parameters.

The lack of consolidated sex-specific reference values represents an important limitation for clinical interpretation. Because GLS thresholds are increasingly used in diagnostic and prognostic decision-making, reliance on pooled reference ranges that do not account for sex differences may lead to misclassification of physiological versus pathological myocardial function. Furthermore, clarifying the relationship between deformation-derived measures and conventional indices such as LVEF is essential to contextualize the physiological meaning of strain measurements.

Therefore, a systematic synthesis of available evidence is warranted. The aim of the present systematic review and meta-analysis was to evaluate GLS values measured by 2D- and 3D-STE in healthy adults and to quantify sex-related differences in myocardial longitudinal deformation. In addition, a complementary meta-analysis of LVEF was performed to contextualize deformation findings relative to conventional measures of global systolic function. By pooling data from contemporary studies including healthy populations, we sought to provide consolidated reference estimates and clarify the magnitude and consistency of sex-specific variation in normal myocardial longitudinal mechanics.

## 2. Materials and Methods

This systematic review and meta-analysis were performed following the Preferred Reporting Items for Systematic Reviews and Meta-Analyses (PRISMA) guidelines [20]. The completed PRISMA checklist is available in the Supplementary Materials (Supplementary Material File S1).

The review protocol was registered in the INPLASY database (registration number: INPLASY202630007; registration date: 2 March 2026; Supplementary Material File S2).

### 2.1. Search Strategy

A comprehensive literature search was independently performed by two investigators to identify all studies reporting GLS values measured in healthy individuals using STE. Electronic databases, including PubMed, Scopus, and EMBASE, were systematically searched from database inception to February 2026. The search strategy combined controlled vocabulary terms and free-text keywords related to myocardial deformation imaging and normative populations. Search terms included combinations of “global longitudinal strain”, “GLS”, “myocardial strain”, “speckle tracking echocardiography”, “2D-STE”, “3D-STE”, “healthy subjects”, “normal population”, and “reference values”. No restrictions were applied regarding language, publication year, or geographic region.

Because the secondary objective of the study was to compare myocardial deformation with conventional systolic function indices, LVEF data were additionally extracted from all eligible studies identified through the GLS-focused search strategy when such information was available. No separate search strategy was performed for LVEF, as the analysis was designed to evaluate complementary physiological information within the same healthy populations.

In addition, reference lists of all eligible articles and relevant review papers were manually screened to identify potentially relevant studies not captured through the electronic search. Discrepancies between reviewers during the screening process were resolved by discussion and consensus, with consultation of a third investigator when required.

### 2.2. Eligibility Criteria

Studies were considered eligible if they had an observational design, including cross-sectional or cohort studies, and evaluated myocardial deformation in healthy adult populations free of known cardiovascular disease. Eligible studies were required to assess GLS using echocardiographic techniques, specifically 2D-STE or 3D-STE, and to provide extractable quantitative GLS data reported as mean ± standard deviation, median with dispersion measures, or in a format suitable for statistical transformation. Studies were additionally required to report sex-specific GLS values or provide sufficient information to allow comparison between women and men.

Studies reporting sex-stratified LVEF values, or providing sufficient information to derive sex-specific LVEF comparisons, were additionally considered eligible for inclusion in the secondary quantitative synthesis aimed at comparing myocardial deformation with conventional systolic function indices.

Only studies evaluating LV-GLS derived from echocardiographic speckle-tracking analysis in human subjects were included. Studies in which myocardial deformation was assessed using cardiac magnetic resonance (CMR)—including feature-tracking CMR, myocardial tagging, strain-encoded imaging (SENC), or other CMR-based strain techniques—were excluded because of fundamental differences in acquisition methodology, tracking algorithms, and modality-specific reference values compared with echocardiographic strain measurements.

For the complementary LVEF analysis, conventional echocardiographic measurements obtained using standard 2D or 3D imaging were considered eligible irrespective of strain vendor or tracking software, given the modality-independent nature of volumetric ejection fraction assessment.

Studies conducted in animal models or experimental preclinical settings were also excluded to ensure physiological comparability and clinical applicability of pooled reference values.

Furthermore, studies primarily focused on right ventricular global longitudinal strain (RV-GLS), left atrial strain parameters (including LASr), or deformation indices other than LV-GLS were not considered eligible.

Studies were excluded if they enrolled mixed populations without separable healthy cohorts, lacked quantitative GLS reporting, used experimental or non-standardized strain methodologies, or provided insufficient data for pooled analysis. Non-original publications, including editorials, conference abstracts, letters, case reports, narrative reviews, and guidelines, were also excluded.

Studies lacking extractable quantitative LVEF data were excluded from the secondary LVEF meta-analysis but remained eligible for inclusion in the primary GLS synthesis.

### 2.3. Study Selection and Data Extraction

Two investigators independently screened all retrieved records by title and abstract, followed by full-text assessment of potentially eligible studies according to predefined inclusion and exclusion criteria. Disagreements regarding eligibility were resolved through discussion and consensus, and when necessary a third reviewer adjudicated disagreements.

Data extraction was independently performed using a standardized data collection form developed a priori. Extracted variables included study characteristics (first author, publication year, country, study design, imaging modality, and sample size).

Demographic and anthropometric parameters included age, sex distribution, body mass index (BMI), body surface area, and cardiovascular risk profile variables when available (including blood pressure values, heart rate, smoking prevalence, serum cholesterol, and glucose levels).

Clinical and conventional echocardiographic variables were systematically collected to characterize cardiac structure and function in healthy populations. These included interventricular septal thickness, posterior wall thickness, left ventricular end-diastolic diameter, relative wall thickness, left ventricular mass index, left ventricular end-diastolic and end-systolic volumes, LVEF, left atrial volume index, and right ventricular structural and functional parameters such as basal right ventricular diameter, tricuspid annular plane systolic excursion (TAPSE), and estimated pulmonary pressure indices when reported.

Sex-specific LVEF values for women and men were extracted whenever available. When studies reported only overall LVEF values or reference limits, additional information allowing the derivation or reconstruction of sex-specific summary statistics was recorded for subsequent quantitative synthesis.

Diastolic function parameters were also extracted, including transmitral E/A ratio and E/e′ ratio.

Strain-derived measures included GLS values, sex-specific GLS estimates for women and men, and additional deformation parameters when available, including global circumferential strain (GCS), global radial strain, right ventricular longitudinal strain indices, and view-specific longitudinal strain measurements derived from apical four-chamber, two-chamber, and three-chamber views. Segmental longitudinal strain values were additionally recorded when reported, including basal, mid-ventricular, and apical strain measurements. For consistency of presentation and to facilitate reader interpretation, GLS and GCS values were expressed throughout tables and meta-analytic analyses as absolute positive values, although originally reported as negative percentages reflecting myocardial shortening. This transformation was applied uniformly across studies without altering relative differences or statistical comparisons.

When numerical values were available only in graphical format, data were extracted using digital plot analysis software. All extracted data were cross-checked for accuracy by both reviewers, and discrepancies were resolved through re-evaluation of the original manuscripts until agreement was achieved.

### 2.4. Methodological Quality Appraisal and Bias Assessment

Methodological quality and risk of bias of included studies were independently evaluated by two reviewers using the National Institutes of Health (NIH) Quality Assessment Tool for Observational Cohort and Cross-Sectional Studies [21]. This tool examines key methodological domains, including study population definition, eligibility criteria, exposure and outcome assessment, consistency of measurement methods, statistical analysis, and completeness of reporting.

Each study was assessed across 14 domains and rated as “Yes”, “No”, “Cannot determine”, “Not reported”, or “Not applicable”, following NIH guidance. Affirmative responses were considered indicative of fulfilled quality criteria, while items deemed not applicable were excluded from score calculation. A summary score was obtained by counting the number of criteria satisfied.

Overall methodological quality was classified according to predefined thresholds: studies fulfilling 9–14 criteria were rated as good quality, those meeting 5–8 criteria as fair quality, and those fulfilling fewer than 5 criteria as poor quality. Final classification incorporated both the numerical score and reviewer judgment regarding key methodological domains.

Any disagreement in domain ratings or overall quality classification was resolved through discussion and re-evaluation of the original manuscripts until consensus was reached. Methodological quality results were summarized using graphical risk-of-bias representations.

### 2.5. Statistical Analysis

To characterize overall physiological distributions across healthy populations, pooled descriptive reference estimates were derived from study-level data. Continuous variables were summarized as weighted medians and interquartile ranges (IQRs). Because most studies reported results as mean ± standard deviation, study-level distributions were approximated assuming normality and weighted according to study sample size. The resulting pooled distributions were used to estimate overall medians and IQRs, enabling the derivation of normative reference ranges at the population level.

The primary objective of the quantitative synthesis was to evaluate sex-related differences in LV-GLS between healthy women and men. In addition, a secondary meta-analysis was performed to assess sex-related differences in LVEF, allowing comparison between myocardial deformation indices and conventional volumetric measures of systolic function.

Comparative meta-analyses were performed using random-effects models to account for anticipated clinical and methodological heterogeneity across studies. Effect sizes for continuous outcomes were expressed as standardized mean differences (SMDs) with corresponding 95% confidence intervals. SMDs were selected to standardize effect estimates across studies with potentially different dispersion profiles and measurement variability; however, given that GLS is uniformly expressed as a percentage, this approach may reduce the immediate clinical interpretability of the results in absolute terms.

When studies reported continuous variables as medians with dispersion measures, means and standard deviations were estimated using validated statistical conversion methods and used exclusively for meta-analytic pooling. For studies reporting LVEF reference limits without explicit dispersion measures, approximate means and standard deviations were reconstructed assuming normally distributed values. These transformations were applied using established methods but should be interpreted as approximations rather than exact reconstructions of original data distributions. A random-effects model based on the DerSimonian–Laird method was selected a priori.

Between-study heterogeneity was quantified using Cochran’s Q statistic and the I^2^ index, with values of approximately 25%, 50%, and 75% interpreted as low, moderate, and high heterogeneity, respectively.

Pre-specified subgroup analyses were conducted according to imaging modality. For GLS analyses, studies were stratified according to speckle-tracking dimensionality (2D-STE versus 3D-STE). For LVEF analyses, subgroup comparisons were performed according to echocardiographic acquisition technique (2D versus 3D echocardiography). Separate random-effects models were constructed for each subgroup, and between-subgroup differences were evaluated using the Q-test for subgroup interaction.

Publication bias and small-study effects were assessed by visual inspection of funnel plots and formally evaluated using Egger’s regression asymmetry test.

Meta-regression analyses were subsequently performed to explore potential sources of between-study heterogeneity. The following study-level covariates were included a priori: percentage of female participants, mean age, BMI, systolic blood pressure (SBP), heart rate (HR), echocardiographic software vendor (non-GE vs. GE), acquisition frame rate, and ASE segmentation model (non-18 vs. 18-segment model). Regression coefficients (β), standard errors, 95% confidence intervals, and two-sided *p*-values were calculated for each moderator variable.

Sensitivity analyses were performed using a leave-one-out approach to evaluate the robustness of pooled estimates for both GLS and LVEF analyses.

All statistical analyses were performed using Comprehensive Meta-Analysis software (version 3.0; Biostat, Englewood, NJ, USA). All tests were two-tailed, and a *p*-value < 0.05 was considered statistically significant.

## 3. Results

### 3.1. Study Identification and Selection Process

The systematic literature search identified 1234 records. After removal of duplicates, 1132 articles underwent screening, of which 42 were assessed for full-text eligibility. Ultimately, 32 studies [22,23,24,25,26,27,28,29,30,31,32,33,34,35,36,37,38,39,40,41,42,43,44,45,46,47,48,49,50,51,52,53] met the inclusion criteria and were included in the systematic review, and 28 [23,24,26,27,29,30,31,32,33,34,35,36,37,38,39,41,42,43,44,45,46,47,48,49,50,51,52,53] provided sex-stratified GLS data suitable for meta-analysis.

**Figure 1:**
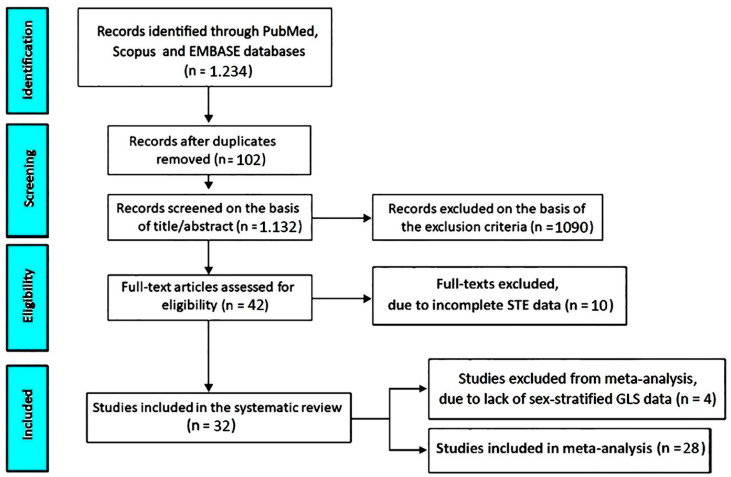
PRISMA flow diagram of the study selection process. GLS, global longitudinal strain. STE, speckle-tracking echocardiography.

The study selection process is summarized in Figure 1 according to PRISMA recommendations.

### 3.2. Study Selection and Overview of Included Studies

A total of 32 studies published between 2009 and 2026 were included in the systematic review and quantitative synthesis, comprising 19,157 healthy individuals recruited across diverse geographic regions, including Asia, Europe, North and South America, Africa, and several multinational collaborative cohorts. The included investigations encompassed a wide range of study settings, from single-center physiological reference cohorts to large multicenter and population-based studies designed to establish normative myocardial deformation values. Detailed characteristics of included studies are summarized in Table 1.

The majority of investigations adopted prospective observational designs, including community-based and population-derived cohorts, while a smaller proportion consisted of retrospective analyses restricted to healthy participants.

Speckle-tracking echocardiography was performed using both 2D and 3D techniques. Two-dimensional STE represented the predominant imaging modality, whereas 3D-STE was primarily applied in multicenter validation and methodological studies evaluating volumetric strain analysis. Image acquisition and analysis were conducted using multiple vendor platforms and post-processing software packages, reflecting real-world technological heterogeneity and the progressive evolution of strain imaging across study periods.

Frame rate acquisition varied according to imaging modality and equipment generation. Studies using 2D-STE generally employed high temporal resolution acquisitions consistent with recommended ranges for longitudinal strain analysis, whereas lower temporal resolution was reported in 3D-STE datasets due to intrinsic limitations of volumetric imaging. Despite variability in acquisition parameters, most studies adhered to guideline-recommended image quality criteria to ensure adequate speckle tracking and reliable GLS quantification.

Left ventricular strain analysis was performed using standardized segmentation models, most commonly 18- or 16-segment approaches, with only minor methodological variation across studies.

Chronologically, the included literature illustrates the transition of STE from early feasibility investigations toward large international collaborative studies employing increasingly standardized acquisition protocols and analytic frameworks, supporting the robustness and contemporary applicability of the pooled dataset.

### 3.3. Population Demographic and Clinical Characteristics

Pooled demographic and clinical characteristics of the included healthy populations are summarized in Table 2.

Overall, the analyzed cohorts demonstrated a consistent demographic and cardiovascular risk profile compatible with physiologically normal populations across diverse geographic regions and study settings. Anthropometric measures, hemodynamic parameters, and available metabolic variables collectively indicated the absence of systematic cardiovascular risk enrichment or structural remodeling patterns within the pooled dataset.

Despite variability in reporting completeness among individual studies, the available data consistently supported the inclusion of participants with preserved cardiovascular health and balanced sex representation. Lifestyle and metabolic indicators, when reported, were similarly aligned with reference population characteristics.

### 3.4. Conventional Echocardiographic Parameters

Conventional echocardiographic measurements across included studies consistently confirmed structurally and functionally normal cardiac phenotypes in the analyzed populations. A comprehensive summary of pooled structural and functional echocardiographic parameters is presented in Table 3.

Left ventricular geometry demonstrated homogeneous characteristics across cohorts, with wall thickness, chamber size, and mass indices collectively indicating the absence of remodeling patterns suggestive of pressure or volume overload. The distribution of relative wall thickness further supported a predominance of normal ventricular geometry, without systematic evidence of concentric or eccentric remodeling among healthy participants.

Indices of diastolic function were uniformly preserved, reflecting normal ventricular relaxation and filling pressures. The overall pattern of transmitral inflow and tissue Doppler parameters was consistent with physiological age-related variation rather than pathological diastolic dysfunction. Volumetric measurements likewise showed balanced ventricular dimensions, supporting adequate preload conditions and reinforcing the representativeness of the included cohorts as reference populations.

Global systolic performance assessed by LVEF remained within expected physiological limits across studies, confirming preserved contractile function in the pooled healthy population. Sex-stratified descriptive estimates demonstrated slightly higher LVEF values in women compared with men, although the magnitude of this difference appeared modest and largely overlapping between sexes.

Similarly, left atrial size and right ventricular structural and functional indices fell within normal reference ranges, indicating the absence of chronic pressure elevation or subclinical cardiopulmonary disease.

Collectively, these findings confirm that myocardial deformation measurements were derived from populations with normal cardiac structure and hemodynamics, thereby providing an appropriate physiological framework for interpretation of strain-derived parameters and for subsequent comparison between deformation-based and volumetric indices of systolic function.

### 3.5. Global and Regional Myocardial Strain in Healthy Individuals

Myocardial deformation parameters derived from STE were reported across included studies, enabling comprehensive characterization of both global and regional myocardial mechanics in healthy adults.

A pooled descriptive summary of strain-derived indices is presented in Table 4.

Global longitudinal strain demonstrated a relatively narrow physiological distribution despite substantial diversity in geographic origin, imaging platforms, and acquisition protocols, supporting the robustness of deformation imaging as a marker of myocardial systolic performance under normal physiological conditions.

Sex-specific analyses revealed systematically higher longitudinal deformation in women compared with men, a finding observed across the majority of cohorts and independent of imaging modality. The stability of this difference across heterogeneous study designs suggests that sex-related variation reflects intrinsic physiological determinants rather than methodological artifacts.

Beyond global longitudinal deformation, studies reporting additional strain components confirmed preservation of multidirectional myocardial mechanics. Circumferential and radial strain indices demonstrated coherent patterns consistent with normal myocardial contractility, supporting the concept that longitudinal strain differences occur within an otherwise balanced mechanical profile rather than reflecting isolated functional alterations.

Regional analyses consistently demonstrated the characteristic physiological gradient of longitudinal deformation from the ventricular base toward the apex. This progressive increase in strain magnitude across myocardial levels was reproducible across studies and imaging platforms, reflecting known variations in myocardial fiber orientation and regional wall stress distribution. The preservation of this gradient across diverse populations further supports the internal validity of pooled strain estimates.

Among studies reporting view-specific measurements, longitudinal strain values derived from different apical imaging planes showed close agreement, indicating good inter-view consistency when standard acquisition protocols are applied. Minor variability between views likely reflects differences in myocardial visualization and tracking geometry rather than true regional functional disparities.

### 3.6. Meta-Analysis of Sex Differences in Global Longitudinal Strain

**Figure 2:**
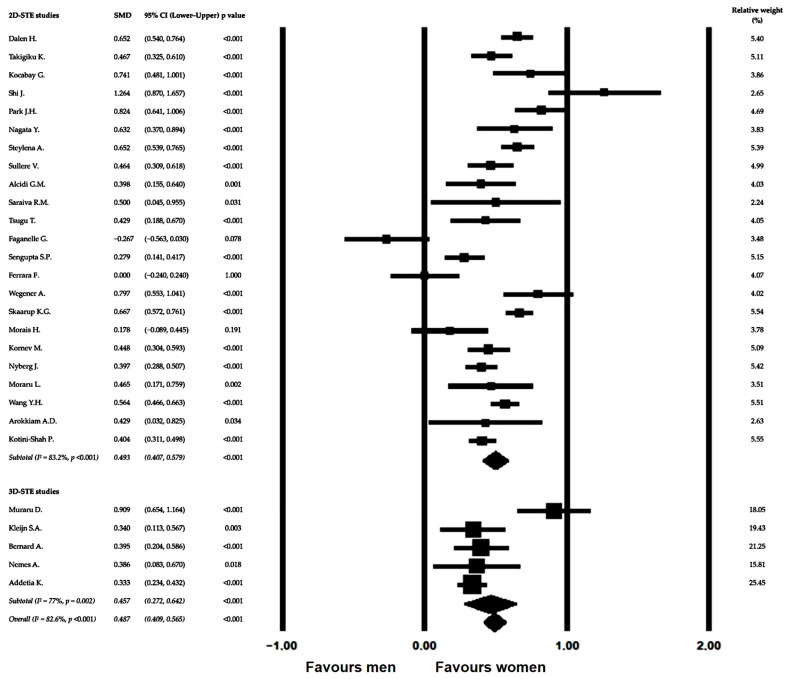
Forest plot of sex-related differences in GLS in healthy individuals, with subgroup analysis of 2D-STE [23,24,26,30,31,33,34,35,36,37,38,39,41,43,44,45,47,48,49,50,51,52,53] and 3D-STE [27,29,32,42,46] studies.

The comparative analysis included 28 studies providing sex-stratified GLS data in healthy populations assessed by STE. The distribution of individual study effect sizes and their corresponding confidence intervals is presented in the forest plot (Figure 2).

Visual inspection of the plot demonstrates a consistent pattern across studies, with most effect estimates favoring higher GLS values in women compared with men. Although variability in effect magnitude was observed, the direction of association was largely uniform, with only a small number of studies showing neutral effects and none demonstrating significantly higher GLS values in men.

Using a random-effects model to account for anticipated clinical and methodological heterogeneity, pooled analysis confirmed significantly higher GLS values in women, with a SMD of 0.487 (95% CI 0.409–0.565; *p* < 0.001), indicating a moderate and statistically robust sex-related difference in longitudinal myocardial deformation.

Subgroup analyses according to imaging modality showed consistent findings across techniques. The pooled SMD was 0.493 (95% CI 0.407–0.579) among studies using 2D-STE and 0.457 (95% CI 0.272–0.642) among studies using 3D-STE. No significant between-subgroup difference was observed (Q = 0.121, *p* = 0.727), indicating that the observed sex-related differences were independent of imaging modality.

Between-study heterogeneity was substantial across the included investigations. Under the fixed-effect model, overall heterogeneity was high, with an I^2^ value of 82.7% (Q = 155.695, *p* < 0.001), indicating that a large proportion of total variability in effect estimates was attributable to true between-study differences rather than sampling error. Similar levels of heterogeneity were observed within imaging modality subgroups, with I^2^ values of 83.2% for studies using 2D-STE and 77.0% for those using 3D-STE.

Variance component analysis demonstrated comparable between-study dispersion across subgroups, with an estimated between-study variance (τ^2^) of approximately 0.032 and τ values around 0.18, supporting the presence of moderate-to-high residual heterogeneity despite consistent effect direction. These findings justified the a priori adoption of random-effects modeling for pooled analyses.

Importantly, although statistical heterogeneity was elevated, the direction of the observed effect remained highly consistent across studies, as illustrated in the forest plot. The vast majority of individual effect estimates favored higher GLS values in women, suggesting that heterogeneity primarily reflected differences in effect magnitude rather than conflicting associations between studies.

Furthermore, subgroup comparison according to imaging modality did not demonstrate significant between-group heterogeneity (Q_between = 0.121, *p* = 0.727), indicating that imaging dimensionality was not a major contributor to variability in pooled effect estimates.

Overall, these results indicate substantial quantitative heterogeneity but strong qualitative consistency of findings, supporting the robustness and generalizability of the observed sex-related differences in GLS.

**Figure 3:**
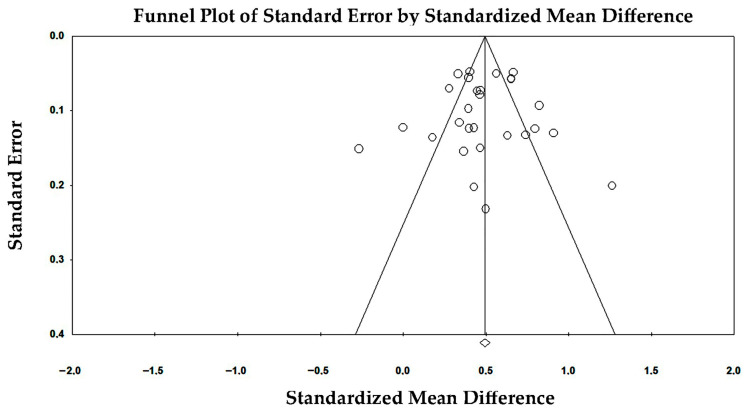
Funnel plot assessing publication bias in studies evaluating sex differences in GLS.

Potential publication bias and small-study effects were evaluated through visual inspection of funnel plot symmetry and formal statistical testing using Egger’s regression asymmetry test. The funnel plot (Figure 3) displays the relationship between study effect size and standard error for the included investigations.

Visual inspection demonstrated an approximately symmetrical distribution of studies around the pooled effect estimate, without clear evidence of asymmetry or clustering suggestive of selective reporting or small-study effects.

Most studies were distributed within the expected triangular region defined by sampling variability, with larger studies concentrated near the pooled estimate and smaller studies showing wider dispersion, consistent with expected statistical behavior. No systematic absence of studies in specific regions of the plot was observed.

Egger’s regression analysis further supported the absence of publication bias. The regression intercept was −0.269 (standard error 1.092), with a non-significant two-tailed *p*-value of 0.81, indicating no statistically detectable funnel plot asymmetry. The 95% confidence interval of the intercept crossed zero (−2.51 to 1.98), reinforcing the lack of evidence for small-study effects.

Overall, both graphical and statistical assessments suggested a low likelihood of publication bias influencing the pooled estimates.

To investigate potential sources of between-study heterogeneity, random-effects meta-regression analyses were performed using predefined study-level demographic, clinical, and methodological covariates (Table 5).

The evaluated moderators included percentage of female participants, mean age, BMI, SBP, HR, echocardiographic software vendor, acquisition frame rate, and the segmentation model adopted for strain analysis according to ASE recommendations.

Overall, meta-regression did not identify any significant moderators of the observed effect size. Neither population-related variables nor technical imaging characteristics were associated with meaningful variation in GLS sex differences across studies. In particular, demographic and physiological parameters reflecting baseline population characteristics did not influence the magnitude of the pooled effect, and methodological factors related to image acquisition and post-processing similarly failed to explain between-study variability.

These findings suggest that the higher GLS values observed in women were consistently reproduced across heterogeneous clinical settings, imaging platforms, and analytical approaches. Consequently, the detected sex-related difference appears to represent a stable physiological phenomenon rather than an association driven by specific study characteristics or technical factors.

Sensitivity analyses were conducted using a leave-one-out approach to evaluate the robustness of pooled effect estimates. Sequential exclusion of individual studies resulted in minimal variation in the pooled SMD, with recalculated estimates remaining tightly clustered around the primary pooled value.

Across all iterations, pooled SMD values ranged narrowly and consistently remained statistically significant (all *p* < 0.001), indicating that no single study exerted disproportionate influence on the overall results. Within subgroup analyses, only small oscillations of effect size were observed. Among studies using 2D-STE, recalculated pooled SMD estimates showed minimal variation around the original subgroup effect, remaining within a narrow range of approximately 0.47 to 0.51 following sequential study removal.

Similarly, in the subgroup comprising the five studies using 3D-STE, pooled estimates demonstrated limited fluctuation, with SMD values consistently confined to an interval of approximately 0.47 to 0.49 across leave-one-out iterations. Confidence intervals showed only minor changes after exclusion of individual studies, and both the direction and magnitude of the sex-related GLS difference were preserved throughout all analyses.

These findings confirm the stability and reliability of the pooled estimate and demonstrate that the observed association was not driven by outlier studies or individual datasets.

### 3.7. Meta-Analysis of Sex Differences in Left Ventricular Ejection Fraction

**Figure 4:**
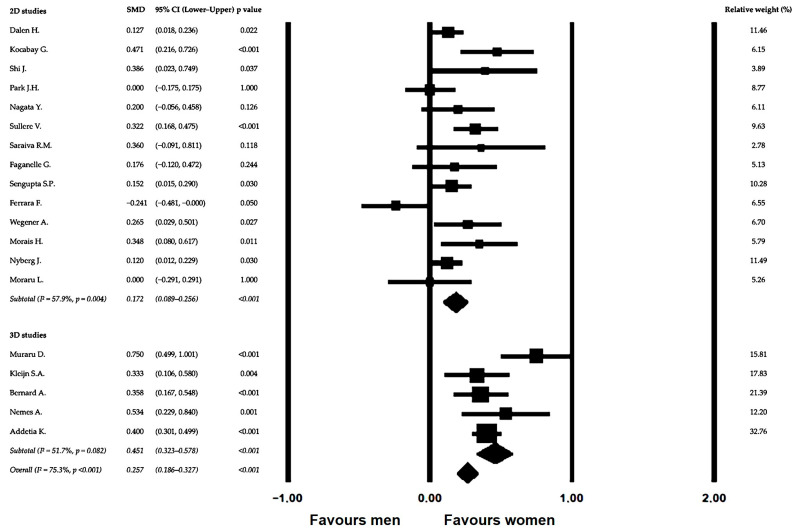
Forest plot of sex-related differences in LVEF in healthy individuals, with subgroup analysis of 2D-STE [23,26,30,31,33,35,37,39,41,43,44,47,49,50] and 3D-STE [27,29,32,42,46] studies.

To contextualize the observed sex-related differences in myocardial deformation, an additional meta-analysis was conducted to evaluate sex-related differences in LVEF measured by conventional echocardiography in healthy populations. The distribution of individual study effect sizes and corresponding confidence intervals is illustrated in the forest plot (Figure 4).

Visual inspection demonstrated that most study estimates were centered close to the null effect, with only modest deviations favoring slightly higher LVEF values in women.

Using a random-effects model, pooled analysis demonstrated a small but statistically significant difference between sexes, with an overall SMD of 0.257 (95% CI 0.186–0.327; *p* < 0.001). The magnitude of this effect was notably smaller than that observed for GLS, indicating limited sex-related separation in global systolic performance despite consistent differences in myocardial deformation.

Subgroup analyses according to imaging dimensionality showed comparable findings across modalities. The pooled SMD was 0.172 (95% CI 0.089–0.256) among studies using 2D echocardiography and 0.451 (95% CI 0.323–0.578) among studies using 3D echocardiography, with significant between-subgroup variability (Q_between = 12.763, *p* < 0.001), suggesting a modest influence of imaging technique on effect magnitude.

Between-study heterogeneity was moderate-to-high, with an overall I^2^ value of 75.35% (Q = 73.032, *p* < 0.001). Similar heterogeneity levels were observed within modality-specific subgroups (2D-STE I^2^ = 57.89%; 3D-STE I^2^ = 51.66%). Despite quantitative heterogeneity, the direction of effect remained consistent across studies, with no evidence of systematically higher LVEF values in men.

**Figure 5:**
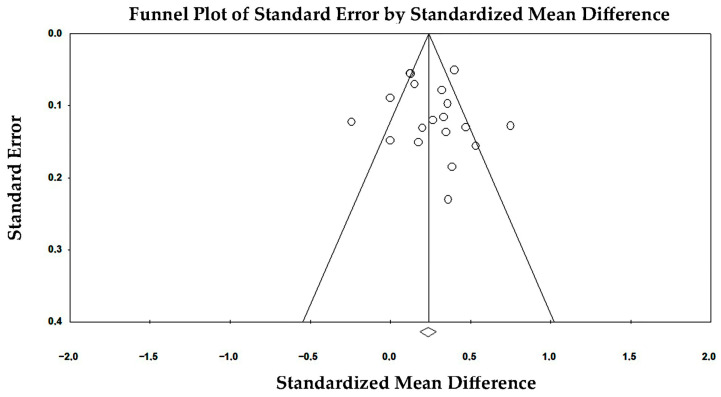
Funnel plot assessing publication bias in studies evaluating sex differences in LVEF.

Potential publication bias was evaluated using funnel plot inspection (Figure 5) and Egger’s regression test.

The funnel plot demonstrated an approximately symmetrical distribution of studies around the pooled estimate without clear evidence of small-study effects. Egger’s regression intercept was 0.721 (SE 1.162), with a non-significant two-tailed *p*-value of 0.54, indicating the absence of statistically detectable publication bias.

Random-effects meta-regression analyses were performed to explore potential moderators of sex-related differences in LVEF, including demographic variables and methodological imaging characteristics. Results are summarized in Table 6.

None of the evaluated covariates demonstrated a significant association with effect size, suggesting that observed sex-related differences in LVEF were not driven by population characteristics or technical acquisition parameters.

Sensitivity analyses using a leave-one-out approach confirmed the robustness of pooled estimates. Sequential exclusion of individual studies resulted in minimal variation in pooled SMD values, which remained tightly clustered around the primary estimate. Across all studies, recalculated pooled effects ranged from approximately 0.228 to 0.279, with persistent statistical significance across all iterations (all *p* < 0.001).

When examined by imaging modality, slightly wider oscillations were observed among studies using 2D-STE, with pooled SMD estimates varying approximately between 0.244 and 0.279 following sequential study removal. In contrast, studies employing 3D-STE demonstrated greater stability, with pooled estimates remaining confined to a narrower interval of approximately 0.243 to 0.251.

Confidence intervals showed only minor fluctuations after exclusion of individual datasets, and both the direction and magnitude of the sex-related difference in LVEF were preserved throughout all iterations, indicating that no single study exerted disproportionate influence on the overall results.

### 3.8. Methodological Quality Assessment

The methodological quality of included studies was evaluated using the NIH Quality Assessment Tool for Observational Cohort and Cross-Sectional Studies. Detailed domain-level assessments are summarized in Supplementary Material File S3 [22,23,24,25,26,27,28,29,30,31,32,33,34,35,36,37,38,39,40,41,42,43,44,45,46,47,48,49,50,51,52,53].

Overall study quality was high. The majority of investigations fulfilled key methodological criteria, including clearly defined study populations, standardized exposure and outcome assessment, and consistent echocardiographic measurement methodologies. Most studies were classified as good quality, while a smaller proportion met criteria for fair quality, primarily due to incomplete reporting of certain methodological domains rather than major design limitations.

The traffic-light risk-of-bias visualization (Supplementary Material File S4) [22,23,24,25,26,27,28,29,30,31,32,33,34,35,36,37,38,39,40,41,42,43,44,45,46,47,48,49,50,51,52,53] illustrates domain-level assessments across individual studies, demonstrating generally low risk of bias in population definition, outcome measurement, and statistical analysis domains. Areas most frequently rated as unclear or not reported involved sample size justification and blinding procedures, which are commonly underreported in observational imaging studies.

The overall risk-of-bias summary plot (Supplementary Material File S5) [22,23,24,25,26,27,28,29,30,31,32,33,34,35,36,37,38,39,40,41,42,43,44,45,46,47,48,49,50,51,52,53] further confirms the predominance of favorable methodological ratings across domains, with a high proportion of “Yes” assessments and very limited high-risk ratings.

Collectively, these findings indicate that the included studies were methodologically robust, supporting the validity and reliability of the pooled analyses.

## 4. Discussion

### 4.1. Main Findings

In this systematic review and meta-analysis, including more than 19,000 healthy individuals derived from 32 independent studies, we provide a comprehensive synthesis of GLS values obtained by STE and quantify sex-related differences in myocardial deformation under physiological conditions. The present analysis integrates evidence from diverse populations, imaging platforms, and study designs, allowing a robust characterization of normal myocardial longitudinal mechanics.

A complementary evaluation of LVEF was additionally performed to interpret deformation findings in relation to conventional indices of global systolic performance.

Several key findings emerge from this work. First, pooled descriptive estimates demonstrate that GLS values in healthy individuals fall within a relatively narrow physiological range despite substantial differences in geographic origin, demographic characteristics, and acquisition protocols across studies. Conventional echocardiographic parameters consistently confirmed preserved cardiac structure and function, supporting the validity of the included cohorts as representative healthy reference populations. These findings reinforce GLS as a stable marker of myocardial systolic performance under physiological conditions.

Second, women exhibited significantly higher absolute GLS values compared with men, with a moderate but highly consistent pooled effect size across studies. This difference was observed across nearly all included cohorts and remained statistically significant after sensitivity analyses, indicating that sex-related variation represents a reproducible biological phenomenon rather than a study-specific observation. The stability of pooled estimates across leave-one-out analyses further confirms that the observed association was not driven by individual datasets or influential studies.

When interpreted alongside LVEF findings, these results indicate that sex-related differences are expressed more prominently at the level of myocardial deformation than at the level of global ventricular emptying, which remained largely comparable between women and men despite preserved physiological variability.

Third, subgroup analyses according to imaging modality demonstrated comparable effect estimates between 2D- and 3D-STE. The absence of significant interaction between modalities suggests that sex-related differences in GLS are independent of acquisition dimensionality and are preserved across contemporary deformation imaging technologies. Importantly, although between-study heterogeneity was substantial, the direction of effect remained highly consistent across investigations, indicating variability primarily in effect magnitude rather than in biological association.

The comparatively smaller separation observed for LVEF further supports the concept that deformation imaging detects subtler physiological differences that may be partially buffered when ventricular performance is assessed using volumetric indices integrating geometry and loading conditions.

The robustness of the findings is additionally supported by several complementary methodological assessments. Evaluation of publication bias using funnel plot inspection and Egger’s regression test did not demonstrate significant asymmetry, reducing the likelihood that selective reporting influenced pooled estimates. Furthermore, meta-regression analyses incorporating demographic, clinical, and technical covariates failed to identify significant moderators of effect size, suggesting that sex-related GLS differences persist across heterogeneous populations and imaging conditions. Together with the results of heterogeneity analyses, these findings indicate that residual variability is unlikely to be explained by measured study-level characteristics and instead reflects expected physiological and methodological diversity inherent to multicenter imaging research.

Taken together, the combined analyses suggest that sex-related physiological variation primarily influences myocardial contractile mechanics rather than overall pump efficiency, highlighting the complementary but distinct physiological information conveyed by GLS and LVEF.

### 4.2. Physiological Basis of Sex Differences in GLS

The observed sex-related differences in GLS likely reflect intrinsic variations in cardiac morphology, myocardial architecture, and ventricular mechanics rather than pathological alterations in systolic function. Women generally exhibit smaller ventricular volumes, reduced myocardial mass, and distinct ventricular geometry compared with men [54,55]. These anatomical characteristics may favor enhanced longitudinal fiber shortening and lead to higher measured strain values despite comparable LVEF, underscoring the concept that GLS captures myocardial mechanics beyond conventional volumetric indices [56,57].

Longitudinal myocardial deformation primarily reflects subendocardial fiber function, which is particularly sensitive to wall stress and loading conditions [58,59]. Because longitudinal fibers are predominantly located in the subendocardial layer, they are strongly influenced by ventricular geometry and myocardial stress distribution [60]. Sex-related differences in ventricular size and wall stress may therefore modulate strain measurements independently of intrinsic myocardial contractility, providing a physiological explanation for higher GLS values in women. Smaller ventricular cavities, associated with lower radius-to-wall thickness ratios, may allow more efficient longitudinal shortening for a given stroke volume, resulting in greater deformation without necessarily indicating superior systolic performance [61,62].

Hormonal influences likely further contribute to these physiological differences. Estrogen has been associated with improved endothelial function, enhanced nitric oxide bioavailability, reduced myocardial fibrosis, and more favorable myocardial energetic efficiency [63,64,65]. These effects may promote enhanced subendocardial contractile performance and preserve myocardial compliance, thereby facilitating greater longitudinal deformation. Conversely, higher myocardial mass and relatively increased afterload conditions typically observed in men may attenuate longitudinal shortening while preserving overall pump function, reflecting differences in myocardial adaptation rather than dysfunction.

Additionally, sex-specific differences in myocardial fiber orientation, extracellular matrix composition, and ventricular remodeling patterns may influence strain measurements [66]. Variations in collagen turnover, myocardial stiffness, and fiber helicity may alter deformation mechanics even in structurally normal hearts [67,68]. Emerging evidence suggests that sex-related myocardial phenotypes represent distinct physiological adaptations rather than a continuum of the same mechanical profile, which may explain why GLS differences persist across populations, imaging modalities, and analytic platforms.

Importantly, the consistency of findings across multiple independent cohorts and methodological settings supports the interpretation that sex-related GLS variation represents a normal physiological characteristic rather than measurement variability or technical artifact. The reproducibility of this difference across heterogeneous datasets reinforces the biological plausibility of sex-specific myocardial deformation patterns.

### 4.3. Clinical Implications

The clinical implications of these findings are particularly relevant given the expanding role of GLS in contemporary cardiovascular practice. GLS is increasingly used for early detection of subclinical myocardial dysfunction, risk stratification in cardiomyopathies, and monitoring of cardiotoxic therapies [69,70]. In these contexts, relatively small deviations from reference values may influence diagnostic classification, surveillance strategies, and therapeutic decisions. In contrast, conventional indices such as LVEF showed only modest sex-related differences in the present analysis, suggesting that ventricular pump performance remains largely comparable between sexes under physiological conditions. This distinction highlights that deformation imaging captures physiological variability that may not be reflected by conventional volumetric measures.

The consistent observation of higher GLS values in women indicates that applying uniform reference thresholds may lead to systematic misclassification. Women may appear to have supranormal myocardial function, whereas men may be incorrectly classified as having mildly impaired systolic function despite normal myocardial mechanics. This issue is particularly relevant in borderline clinical settings—such as early cardiomyopathy detection, heart failure with preserved ejection fraction (HFpEF), and cardio-oncology surveillance—where GLS thresholds are often used as decision-support parameters.

From a practical standpoint, these findings suggest that clinicians should interpret GLS values in a sex-specific manner, particularly when values fall near commonly used diagnostic thresholds, where misclassification is more likely to occur.

The adoption of sex-specific GLS reference ranges may therefore improve diagnostic accuracy by reducing both false-positive interpretations in men and false-negative assessments in women. Importantly, these findings do not imply the need for sex-specific LVEF thresholds, but rather emphasize that deformation parameters and volumetric indices provide complementary physiological information.

However, given the variability observed across studies and methodological settings, sex-specific thresholds should be applied with clinical judgment and in conjunction with technical considerations, rather than as rigid cut-off values.

Interpretation of GLS values must also consider technical differences between two- and three-dimensional STE. In routine clinical practice, 2D-STE remains the most widely used modality because of its availability, higher temporal resolution, and extensive validation across disease states [71]. However, it is dependent on optimal imaging planes and may be affected by foreshortening or out-of-plane motion, particularly during serial follow-up where small GLS variations may be clinically relevant.

Three-dimensional STE offers conceptual advantages by analyzing myocardial motion within a volumetric dataset, thereby reducing plane dependency and enabling more comprehensive characterization of myocardial deformation, including parameters such as area or principal strain [9]. Nevertheless, 3D-derived GLS values are generally lower than those obtained with 2D-STE, with reported differences of approximately 4%, indicating that measurements from the two techniques are not interchangeable and require modality-specific reference ranges [72,73,74]. In selected populations, including HFpEF, 3D-STE has also demonstrated incremental prognostic value [75].

Despite these advantages, 3D-STE remains limited by lower temporal resolution and greater sensitivity to image quality limitations and artifacts, resulting in lower feasibility in routine practice compared with 2D imaging [76]. Consequently, absolute GLS values should not be directly compared across modalities, and longitudinal follow-up should ideally be performed using the same imaging technique, vendor, and software version [77].

Overall, the consistent sex-related differences observed across both 2D- and 3D-STE support the adoption of biologically informed interpretation frameworks. Sex-specific GLS reference values may improve diagnostic precision and reduce systematic bias in both clinical practice and research settings. Integrating sex-adjusted thresholds into GLS interpretation, therefore, represents an important step toward more personalized evaluation of myocardial function.

From a clinical perspective, the use of sex-specific GLS interpretation appears particularly relevant in settings characterized by subtle or early myocardial dysfunction, including cardio-oncology surveillance, early-stage cardiomyopathies, and HFpEF, where small deviations from normal values may influence clinical decision-making. In these contexts, sex-adjusted interpretation may help refine risk stratification and reduce diagnostic uncertainty.

Based on the pooled descriptive estimates derived from this meta-analysis, we suggest preliminary sex-specific normal reference ranges for GLS in healthy adults of approximately 18.5–23.0% in women and 17.0–21.5% in men, reflecting the observed central tendency and interquartile distribution of values. These ranges should be considered as clinically informative benchmarks rather than definitive universal thresholds and should be interpreted in the context of imaging methodology, vendor-specific characteristics, and individual patient profiles.

**Figure 6:**
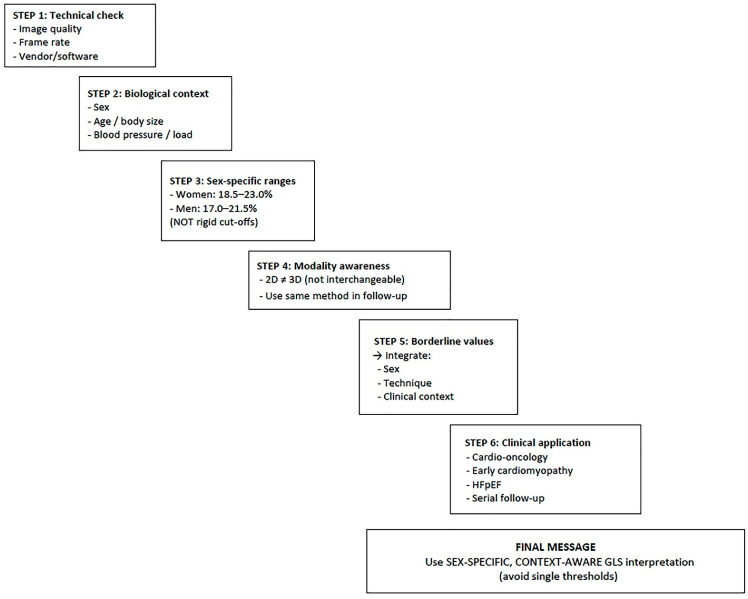
Practical algorithm for sex-specific interpretation of LV-GLS. This schematic framework summarizes a stepwise approach for GLS interpretation in healthy adults, integrating technical, biological, and clinical factors. Initial evaluation includes verification of image quality, acquisition parameters, and imaging modality (2D vs. 3D speckle-tracking echocardiography). GLS values should then be interpreted according to biological context, particularly sex, using sex-specific reference ranges (approximately 18.5–23.0% in women and 17.0–21.5% in men). Additional considerations include modality-specific differences and consistency of imaging techniques during follow-up. In borderline cases, GLS interpretation should incorporate clinical context, including cardio-oncology surveillance, early cardiomyopathy, and heart failure with preserved ejection fraction (HFpEF). Overall, the algorithm emphasizes that GLS values should be interpreted using a sex-specific, context-aware approach rather than applying uniform diagnostic thresholds.

To support clinical implementation, a simplified stepwise interpretative algorithm is provided (Figure 6), highlighting the importance of integrating technical quality, sex, imaging modality, and clinical context when interpreting GLS values, particularly in borderline cases where the risk of misclassification is highest.

### 4.4. Sources of Heterogeneity

Substantial statistical heterogeneity was observed across included studies, which is expected in meta-analyses of physiological imaging parameters derived from heterogeneous populations and evolving imaging methodologies. However, the magnitude of this variability warrants careful clinical interpretation, particularly when deriving reference values intended for use in routine practice. Importantly, heterogeneity in this context reflects the complexity of real-world myocardial deformation assessment rather than the inconsistency of biological findings.

Technical differences represent a major contributor to variability. Variations in vendor-specific tracking algorithms, image acquisition settings, temporal resolution, and post-processing workflows may influence absolute GLS values. Additional sources of variability include differences in segmentation models, software versions, and operator-dependent factors, all of which may significantly affect strain quantification. Although international standardization initiatives have improved inter-vendor comparability [78], residual methodological variability remains an inherent characteristic of strain imaging, particularly across studies conducted over different technological eras.

Population-related factors also likely contributed to heterogeneity. Included studies encompassed diverse ethnic backgrounds, age ranges, anthropometric profiles, and cardiovascular risk characteristics. Differences in body size, arterial load, blood pressure, and autonomic tone may modulate myocardial deformation independently of intrinsic myocardial contractility. Furthermore, variability in inclusion criteria and the definition of “healthy” across studies may have introduced additional heterogeneity related to subclinical or borderline clinical conditions. Physiological heterogeneity across healthy populations therefore represents an expected source of variability rather than methodological bias.

Importantly, despite elevated I^2^ values, the direction of the observed effect remained highly consistent across studies, and sensitivity analyses confirmed stability of pooled estimates. This pattern indicates that heterogeneity primarily reflects variation in effect magnitude rather than conflicting biological associations. Supporting this interpretation, meta-regression analyses failed to identify significant moderators explaining between-study variability, suggesting that the observed sex-related GLS difference is robust across demographic and technical contexts.

Nevertheless, these findings should be interpreted with caution when translating pooled estimates into clinical reference values. The observed variability limits the direct applicability of a single universal threshold and supports a more flexible, context-dependent interpretation of GLS values, taking into account technical settings, vendor-specific characteristics, and patient profile.

Accordingly, the reference ranges proposed in the present analysis should be considered as indicative rather than definitive and primarily useful for framing sex-related differences rather than establishing rigid diagnostic cut-offs. Further prospective standardized studies are needed to refine GLS reference values and improve their generalizability across clinical settings.

### 4.5. Strengths and Limitations

The present study has several important strengths. It represents one of the largest syntheses of GLS measurements in healthy individuals to date and integrates data obtained using both 2D- and 3D-STE across multiple geographic regions and diverse populations. The inclusion of studies performed with different imaging platforms enhances the external validity of the findings and reflects real-world clinical practice. The combined use of pooled descriptive statistics and comparative meta-analysis allowed simultaneous characterization of normative GLS distributions and quantification of sex-related differences within a unified analytical framework. In addition, the application of complementary methodological approaches—including subgroup analyses, heterogeneity assessment, meta-regression, publication bias evaluation, and sensitivity analyses—strengthens the robustness and internal consistency of the conclusions. The inclusion of a complementary quantitative synthesis of LVEF further represents a methodological strength, as it enabled direct comparison between deformation-based and conventional volumetric indices within the same physiological populations, providing additional interpretative context for the observed sex-related differences.

Nevertheless, several limitations should be acknowledged. First, the analysis relied on study-level rather than individual patient data, limiting the ability to adjust for potential confounders such as age distribution, blood pressure, anthropometric characteristics, and subtle differences in cardiovascular risk profiles. Individual participant data meta-analysis would allow more precise modeling of physiological determinants of myocardial deformation but was not feasible given the available literature. Similarly, the LVEF analysis relied on aggregated study-level data, and sex-specific values were not uniformly reported across all studies, requiring reconstruction of summary statistics in selected cases, which may introduce approximation error despite standardized assumptions. In addition, the use of SMDs, while methodologically appropriate for pooling heterogeneous datasets, may reduce the immediate clinical interpretability of GLS differences expressed in percentage units.

Second, methodological variability between vendors and acquisition protocols may have influenced absolute strain values. Despite ongoing standardization efforts, inter-vendor variability remains a recognized limitation of STE, as differences in tracking algorithms, spatial smoothing, and post-processing pipelines may yield systematically different GLS measurements even when analyzing identical image datasets. Although LVEF measurements are generally less sensitive to vendor-specific variability than strain parameters, differences in imaging acquisition, border delineation techniques, and geometric assumptions across studies may still have contributed to variability in volumetric estimates.

Third, GLS remains partially load-dependent, and differences in physiological loading conditions across cohorts—including variations in blood pressure, heart rate, preload, and afterload—could not be fully accounted for. Because longitudinal strain reflects subendocardial mechanics, it is particularly sensitive to transient hemodynamic changes, which may introduce variability independent of intrinsic myocardial function [79].

Fourth, the derivation of pooled reference values was based on weighted medians and reconstructed distributions from aggregated study-level data. This approach relies on assumptions of approximate normality and indirect estimation of dispersion measures, which may introduce bias and should be interpreted as providing approximate rather than definitive population-level estimates.

Fifth, the definition of “healthy population” was based on the original study-specific inclusion criteria and was not uniform across studies. Although participants were generally free of overt cardiovascular disease, the potential inclusion of individuals with subclinical or borderline conditions may have contributed to between-study heterogeneity and may influence the derived reference ranges.

Importantly, several intrinsic limitations of STE itself must also be considered. The reproducibility of STE analysis may be affected by operator experience, image acquisition quality, and technical settings, particularly frame rate optimization, which directly influences speckle tracking accuracy and temporal resolution [80,81]. Suboptimal image quality, acoustic window limitations, or inadequate endocardial border delineation may reduce tracking reliability and increase measurement variability. Furthermore, STE measurements are susceptible to inter-observer and intra-observer variability, especially in multicenter studies performed over long time periods with evolving software versions.

**Figure 7:**
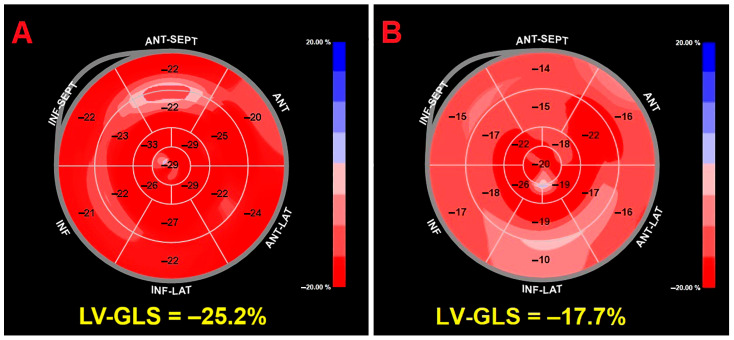
Representative GLS bull’s-eye maps in healthy individuals with different thoracic geometry. Representative examples of left ventricular global longitudinal strain (GLS) obtained by speckle-tracking echocardiography in two healthy women with different thoracic configurations. (**A**) Healthy woman with normal thoracic anatomy showing preserved and homogeneous longitudinal deformation (GLS −25.2%). (**B**) Healthy woman with pectus excavatum demonstrating lower absolute GLS values (GLS −17.7%) despite structurally normal cardiac morphology and preserved ejection fraction, illustrating the potential influence of chest wall geometry on strain measurements.

Extrinsic mechanical factors may also influence deformation measurements. Anterior chest wall deformities, body habitus, and thoracic geometry can alter acoustic windows and myocardial motion tracking, potentially affecting strain estimation despite structurally normal cardiac function [82,83]. Representative examples of GLS bull’s-eye maps obtained in healthy individuals with different thoracic geometries are shown in Figure 7.

Additionally, differences in probe positioning, respiratory motion, and translational cardiac movement may introduce subtle measurement artifacts that are difficult to standardize across studies.

Finally, although formal statistical testing did not demonstrate significant publication bias, the possibility of residual reporting bias cannot be entirely excluded, particularly given the observational nature of the included studies and the likelihood that studies reporting physiologically consistent findings are more frequently published. This consideration applies to both deformation and volumetric analyses, although the consistency observed across independent methodological assessments mitigates concern for systematic bias affecting comparative interpretation.

Overall, these limitations should be interpreted in the context of the strong consistency of results across heterogeneous datasets. While absolute GLS values may vary according to technical and physiological factors, the observed sex-related differences remained stable across analyses, supporting the validity and generalizability of the study conclusions. The parallel evaluation of GLS and LVEF further strengthens this conclusion by demonstrating that sex-related differences remain evident for sensitive markers of myocardial mechanics while global systolic performance appears comparatively preserved, supporting the physiological plausibility of the findings.

## 5. Conclusions

In healthy individuals, GLS demonstrates a relatively narrow physiological distribution together with consistent and clinically relevant sex-related differences, with women exhibiting higher absolute GLS values than men. These differences were observed across heterogeneous populations, imaging platforms, and analytical approaches and remained stable despite substantial between-study variability, supporting their physiological rather than methodological origin.

When interpreted alongside conventional systolic indices, these findings indicate that sex-related variation is more prominently expressed in myocardial deformation than in global ventricular pump performance, which remains largely comparable between women and men under physiological conditions.

The consistency of findings across subgroup analyses, heterogeneity assessment, meta-regression, and sensitivity analyses reinforces the robustness of sex-related variation in myocardial longitudinal deformation. Importantly, the absence of significant moderating effects from demographic or technical factors suggests that biological sex represents an independent determinant of normal myocardial mechanics.

Collectively, these results support the adoption of sex-specific reference values for GLS interpretation and emphasize the need to incorporate biological variability into clinical assessment of myocardial deformation. Integrating sex-adjusted GLS thresholds into routine echocardiographic practice may improve diagnostic accuracy, reduce misclassification of physiological versus pathological myocardial function, and contribute to more personalized cardiovascular evaluation.

Taken together, the combined evaluation of deformation-based and volumetric indices supports a clinical framework in which GLS provides enhanced sensitivity to physiological myocardial differences, while LVEF continues to reflect preserved global systolic function, highlighting the complementary roles of these parameters in cardiovascular assessment.

## Figures and Tables

**Table 1:** Characteristics of included studies [22,23,24,25,26,27,28,29,30,31,32,33,34,35,36,37,38,39,40,41,42,43,44,45,46,47,48,49,50,51,52,53] reporting left ventricular GLS in healthy adults assessed by 2D- and 3D-STE.

Study Name, Publication Year and Country	Design	Method	Software	Mean Frame Rate (fps)	ASE Segm. Model	Size (%Females)	Mean Age (Years)	Mean GLS (%)
Saito K. (2009), Japan [22]	Prospective single-center	3D-STE	Toshiba	20–30	16-segment	46 (13)	29	17
Dalen H. (2010), Norway [23]	Prospective population-based multicenter	2D-STE	GE	44	16-segment	1266 (53.1)	49.1	16.7
Takigiku K. (2012), Japan [24]	Prospective multicenter	2D-STE	GE	62	18-segment	817 (38.5)	36	21.3
Kaku K. (2014), Multinational (Japan, USA) [25]	Prospective multicenter	3D-STE	TomTec	20.7	16-segment	39 (51)	44	19.6
Kocabay G. (2014), Italy [26]	Prospective single-center	2D-STE	GE	50–80	16-segment	247 (56.3)	44	21.5
Muraru D. (2014), Italy [27]	Prospective single-center	3D-STE	GE	NS	17-segment	265 (56.6)	45	19
Xu T.Y. (2014), China [28]	Prospective single-center	3D-STE	GE	20–30	17-segment	230 (40)	51	18.5
Kleijn S.A. (2015), Multinational (8 countries) [29]	Prospective multicenter	3D-STE	Toshiba	20	16-segment	303 (48.5)	42	15.9
Shi J. (2016), China [30]	Prospective single-center	2D-STE	GE	74.2	16-segment	119 (50.4)	48	21.3
Park J.H. (2016), S. Korea [31]	Prospective multicenter	2D-STE	GE	60	18-segment	501 (52.9)	47	20.4
Bernard A. (2017), Multinational (Europe + USA, NORRE) [32]	Prospective multicenter	3D-STE	TomTec	25	17-segment	440 (57.5)	45	21
Nagata Y. (2017), Japan [33]	Retrospective single-center	2D-STE	GE	63	18-segment	235 (50.2)	45	20
Støylen A. (2018), Norway [34]	Prospective population-based single-center	2D-STE	GE	44	18-segment	1266 (52.4)	49.1	16.7
Sullere V. (2018), India [35]	Retrospective single-center	2D-STE	Philips	50–70	17-segment	707 (37.2)	40.6	20
Alcidi G.M. (2018), Italy [36]	Prospective single-center	2D-STE	GE, EchoPAC	70–90	17-segment	266 (51.5)	39.2	22.7
Saraiva R.M. (2019), Brazil [37]	Prospective single-center	2D-STE	GE	>60	18-segment	77 (53.3)	40.4	19
Tsugu T. (2020), Multinational (Europe, NORRE) [38]	Prospective multicenter	2D-STE	GE	63	17-segment	287 (62)	46	21.5
Faganello G. (2020), Italy [39]	Prospective single-center	2D-STE	TomTec	>60	16-segment	176 (51)	47	21.6
Potter E.L. (2021), Australia [40]	Prospective multicenter community-based	2D-STE	Siemens	NS	18-segment	455 (52)	70	18
Sengupta S.P. (2021), India [41]	Prospective multicenter	2D-STE	GE	60–80	18-segment	880 (36.2)	39.7	21
Nemes A. (2021), Hungary [42]	Prospective single-center	3D-STE	Toshiba	NS	16-segment	172 (44.8)	32.7	16.9
Ferrara F. (2021), Italy [43]	Prospective single-center	2D-STE	TomTec	≥50	18-segment	269 (54.3)	43.4	23.1
Wegener A. (2022), Brazil [44]	Prospective single-center	2D-STE	GE	55	18-segment	290 (60)	37	19.9
Skaarup K.G. (2022), Denmark [45]	Prospective population-based single-center	2D-STE	GE	62.8	18-segment	1997 (61.8)	46.3	23.5
Addetia K. (2022), Multinational (15 countries, WASE) [46]	Prospective multicenter international	3D-STE	Philips, GE, Siemens	>20	17-segment	1589 (47.6)	47	21
Morais H. (2022), Angola [47]	Prospective single-center	2D-STE	Mindray DC-70 exp	NS	18-segment	217 (52.5)	41.3	18.3
Kornev M. (2022), Multinational (Russia, Norway) [48]	Prospective population-based multicenter	2D-STE	GE	≥50	18-segment	840 (60.8)	53	20.4
Nyberg J. (2023), Norway [49]	Prospective population-based single-center	2D-STE	GE	71–85	18-segment	1329 (55.7)	57	20
Moraru L. (2024), Romania [50]	Prospective single-center	2D-STE	GE	65	18-segment	200 (35)	37	20
Wang Y.H. (2024), China [51]	Prospective multicenter	2D-STE	Philips	≥50	18-segment	1683 (57)	45	21.4
Arockiam A.D. (2025), USA [52]	Prospective single-center	2D-STE	TomTec, EchoPAC, VVI, Epsilon	52	18-segment	100 (50)	45	17
Kotini-Shah P. (2026), USA [53]	Prospective population-based multicenter	2D-STE	TomTec	NS	12-segment	1818 (57.4)	56.4	17.6

Studies are ordered chronologically. “Method” indicates two-dimensional (2D-STE) or three-dimensional speckle-tracking echocardiography (3D-STE). Frame rate is reported as mean (if available) or as the range/threshold provided by the original study. GLS values are reported as absolute percentages (higher values indicate greater longitudinal shortening). “ASE segm. model” indicates the left ventricular segmentation model used for GLS calculation. 2D, two-dimensional; 3D, three-dimensional; ASE, American Society of Echocardiography; fps, frames per second; GE, General Electric; GLS, global longitudinal strain; NORRE, Normal Reference Ranges for Echocardiography; NS, not specified; STE, speckle-tracking echocardiography; USA, United States of America; VVI, velocity vector imaging; WASE, World Alliance Societies of Echocardiography.

**Table 2:** Weighted pooled demographic, anthropometric, and clinical characteristics of healthy populations included in the systematic review.

Parameter	Weighted Median	Weighted IQR (Q1–Q3)	Studies Included	Size (n)
% females	53	49–57	32	19,157
Age (years)	47.5	36.9–58.1	32	19,157
BSA (m^2^)	1.81	1.66–1.95	31	18,656
BMI (Kg/m^2^)	24.6	22.1–27.6	29	17,609
SBP (mmHg)	122.6	113.8–132.2	29	18,123
DBP (mmHg)	74.6	68.2–80.8	29	18,123
HR (bpm)	69.5	61.6–77.3	20	10,532
Total cholesterol (mg/dL)	195	167–228	7	8394
Glucose (mg/dL)	93	83–106	6	7491
Smoking (%)	17.6	5.0–19.6	4	5253

Values are reported as weighted medians and weighted interquartile ranges (IQR; Q1–Q3), calculated according to study sample size. “Studies included” indicates the number of studies contributing data for each variable, whereas “Size (n)” represents the total pooled population analyzed for that parameter. Clinical and laboratory variables refer to baseline characteristics of healthy adult participants included in the analyzed studies. BMI, body mass index; BSA, body surface area; DBP, diastolic blood pressure; HR, heart rate; IQR, interquartile range; SBP, systolic blood pressure.

**Table 3:** Weighted pooled echocardiographic reference values derived from included studies in healthy adult populations.

Parameter	Weighted Median	Weighted IQR (Q1–Q3)	Studies Included	Size (n)
IVS (mm)	8.9	7.7–10.0	12	5254
PW (mm)	8.8	7.5–10.1	11	5054
LVEDD (mm)	46.7	43.1–50.3	12	5131
RWT	0.37	0.31–0.43	13	6613
LVMi (g/m^2^)	76.0	64.3–88.8	16	6009
E/A	1.5	1.2–1.8	14	6117
E/e′	6.9	5.5–8.5	13	5397
LVEDV (mL)	100	78.8–122.0	29	11,374
LVESV (mL)	39.5	29.9–50.4	28	10,494
LVEF (%)	61.1	57.2–65.1	31	14,211
LVEF females (%)	63.3	59–71	19	4595
LVEF males (%)	62.0	58.5–68.2	19	4717
LAVi (mL/m^2^)	20.9	16.9–25.3	9	4973
RV basal diameter (mm)	28.1	25.0–31.9	4	2143
TAPSE (mm)	22.5	20.6–24.5	5	2133
TRV/sPAP (mm/mmHg)	22.8	19.4–26.1	3	1053

Values are presented as weighted medians and weighted interquartile ranges (IQR; Q1–Q3) calculated according to study sample size. “Studies included” indicates the number of studies contributing data for each parameter, whereas “Size (n)” represents the total pooled population analyzed for that specific variable. Measurements refer to standard transthoracic echocardiographic parameters obtained in healthy adults. Sex-specific values are reported where available. E/A, ratio of early (E) to late (A) diastolic transmitral flow velocity; E/e′, ratio of early transmitral flow velocity to early diastolic mitral annular velocity; IQR, interquartile range; IVS, interventricular septal thickness; LAVi, left atrial volume index; LVEDD, left ventricular end-diastolic diameter; LVEDV, left ventricular end-diastolic volume; LVEF, left ventricular ejection fraction; LVESV, left ventricular end-systolic volume; LVMi, left ventricular mass index; PW, posterior wall thickness; RWT, relative wall thickness; RV, right ventricle; sPAP, systolic pulmonary artery pressure; TAPSE, tricuspid annular plane systolic excursion; TRV, tricuspid regurgitation velocity.

**Table 4:** Weighted pooled reference values of myocardial strain parameters derived from included studies in healthy adult populations.

Parameter	Weighted Median	Weighted IQR (Q1–Q3)	Studies Included	Size (n)
GLS (%)	20.3	17.8–22.5	32	19,157
GLS females (%)	20.8	18.4–23.1	28	9332
GLS males (%)	19.4	17.0–21.6	28	8912
4C LS (%)	21.4	19.4–23.4	5	2984
2C LS (%)	21.8	19.8–23.7	5	2984
3C LS (%)	21.0	18.8–23.2	5	2984
Basal LS (%)	17.9	15.4–20.4	17	6716
Mid LS (%)	19.7	17.5–21.8	17	6716
Apical LS (%)	21.9	18.5–25.0	17	6716
GCS (%)	28.7	23.2–32.8	17	8039
GRS (%)	45.7	37.9–54.3	13	4328
RV-GLS (%)	22.0	20.0–24.0	1	77
RV-FWLS (%)	24.0	20.6–27.4	1	77

Values are expressed as weighted medians and weighted interquartile ranges (IQR; Q1–Q3), calculated according to study sample size. “Studies included” indicates the number of studies contributing data for each strain parameter, while “Size (n)” represents the total pooled population analyzed for that variable. Strain measurements were obtained using speckle-tracking echocardiography and include global, chamber-specific, and segmental longitudinal strain, as well as circumferential and radial strain indices. Sex-specific values are reported where available. 2C, two-chamber view; 3C, three-chamber view; 4C, four-chamber view; FWLS, free-wall longitudinal strain; GCS, global circumferential strain; GLS, global longitudinal strain; GRS, global radial strain; IQR, interquartile range; LS, longitudinal strain; RV, right ventricular.

**Table 5:** Random-effects meta-regression analysis exploring potential moderators of sex-related differences in global longitudinal strain.

Covariate	Coefficient	Standard Error	95% Lower	95% Upper	*p*-Value
Intercept	5.110	3.546	–1.840	12.060	0.150
Female proportion (%)	0.004	0.008	–0.013	0.020	0.672
Mean age (years)	0.025	0.018	–0.010	0.060	0.167
BMI (Kg/m^2^)	0.051	0.068	–0.082	0.184	0.454
SBP (mmHg)	–0.047	0.030	–0.105	0.011	0.115
HR (bpm)	–0.010	0.017	–0.043	0.023	0.555
Software vendor (non-GE vs. GE)	–0.526	0.333	–1.179	0.127	0.115
Frame rate (frames/s)	–0.009	0.011	–0.030	0.012	0.398
ASE segmentation model (non-18 vs. 18 segments)	–0.202	0.189	–0.572	0.167	0.283

Random-effects meta-regression results evaluating the association between predefined study-level covariates and the standardized mean difference in global longitudinal strain between sexes. Regression coefficients represent the estimated change in effect size associated with each covariate. Confidence intervals (95% CI) and corresponding *p*-values are reported for each model parameter. The reference categories were GE software vendor and the 18-segment ASE left ventricular model. ASE, American Society of Echocardiography; BMI, body mass index; bpm, beats per minute; CI, confidence interval; GE, General Electric; HR, heart rate; SBP, systolic blood pressure.

**Table 6:** Random-effects meta-regression analysis of potential moderators of sex-related differences in left ventricular ejection fraction.

Covariate	Coefficient	Standard Error	95% Lower	95% Upper	*p*-Value
Intercept	2.252	2.261	–2.179	6.683	0.319
Female proportion (%)	0.008	0.009	–0.009	0.026	0.356
Mean age (years)	–0.010	0.014	–0.037	0.016	0.446
BMI (Kg/m^2^)	0.009	0.053	–0.096	0.113	0.871
SBP (mmHg)	–0.012	0.017	–0.046	0.021	0.471
HR (bpm)	–0.010	0.020	–0.048	0.029	0.623
Software vendor (non-GE vs. GE)	0.029	0.121	–0.207	0.266	0.809

Random-effects meta-regression results assessing the association between predefined study-level covariates and the standardized mean difference in left ventricular ejection fraction between sexes. Regression coefficients represent the estimated change in effect size associated with each covariate. Ninety-five percent confidence intervals (95% CI) and corresponding *p*-values are reported for all model parameters. The reference category for software vendors was GE.

## Data Availability

Data extracted from included studies will be publicly available on Zenodo (https://zenodo.org).

## Supplementary Materials

The following supporting information can be downloaded at: https://www.mdpi.com/article/10.3390/jcm15082859/s1, Supplementary Materials File S1: PRISMA 2020 checklist; Supplementary Materials File S2: The full INPLASY record (https://inplasy.com/inplasy-2026-3-0007/, accessed on 2 March 2026) [84]; Supplementary Materials File S3: NIH Quality Assessment of included studies [22,23,24,25,26,27,28,29,30,31,32,33,34,35,36,37,38,39,40,41,42,43,44,45,46,47,48,49,50,51,52,53]; Supplementary Materials File S4: Traffic-light plot of risk-of-bias assessment across included studies [22,23,24,25,26,27,28,29,30,31,32,33,34,35,36,37,38,39,40,41,42,43,44,45,46,47,48,49,50,51,52,53]; Supplementary Materials File S5: Overview of risk-of-bias judgments across included studies [22,23,24,25,26,27,28,29,30,31,32,33,34,35,36,37,38,39,40,41,42,43,44,45,46,47,48,49,50,51,52,53].
