# Supplementary material for: Sex-Related Differences in Myocardial Deformation and Systolic Function in Healthy Individuals: A Systematic Review and Meta-Analysis of Global Longitudinal Strain and Left Ventricular Ejection Fraction
Source: J Clin Med. 2026 Apr 9;15(8):2859. doi: 10.3390/jcm15082859 (PMC13115635; doi:10.3390/jcm15082859)
Supplement: Supplementary file 1 [file jcm-15-02859-s001.zip › Supplementary Materials S2.pdf]

International Platform of Registered Systematic Review and Meta-analysis Protocols

INPLASY

INPLASY202630007

doi: 10.37766/inplasy2026.3.0007

Received: 2 March 2026

Published: 2 March 2026

Corresponding author:
Andrea Sonaglioni

sonaglioniandrea@gmail.com

Author Affiliation:
IRCCS MultiMedica.

Sex-Related Differences in Myocardial Deformation and Systolic Function in Healthy Individuals: A Systematic Review and Meta-Analysis of Global Longitudinal Strain and Left Ventricular Ejection Fraction

Sonaglioni, A; Gramaglia, GF; Nicolosi, GL; Baravelli, M; Lombardo, M.

ADMINISTRATIVE INFORMATION

Support - Ministero della Salute.

Review Stage at time of this submission - Completed but not published.

Conflicts of interest - None declared.

INPLASY registration number: INPLASY202630007

Amendments - This protocol was registered with the International Platform of Registered Systematic Review and Meta-Analysis Protocols (INPLASY) on 2 March 2026 and was last updated on 2 March 2026.

INTRODUCTION

Review question / Objective The aim of the present systematic review and meta-analysis was to evaluate global longitudinal strain (GLS) values measured by two-dimensional (2D) and three-dimensional (3D) speckle-tracking echocardiography (STE) in healthy adults and to specifically quantify sex-related differences in GLS between women and men. In addition, a complementary meta-analysis of left ventricular ejection fraction (LVEF) was performed to contextualize deformation findings relative to conventional measures of global systolic performance and to assess whether sex-related physiological differences are similarly expressed across deformation and volumetric indices. By pooling data from contemporary studies including healthy populations, we sought to provide consolidated reference estimates and clarify the magnitude and consistency of sex-specific variation in normal myocardial longitudinal function.The aim of the present systematic review and meta-analysis was to evaluate global longitudinal strain values measured by 2D and 3D speckle-tracking echocardiography in healthy adults and to specifically quantify sex-related differences in GLS between women and men. By pooling data from contemporary studies including healthy populations, we sought to provide consolidated reference estimates and clarify the magnitude and consistency of sex-specific variation in normal myocardial longitudinal function.

Rationale The lack of consolidated sex-specific normative GLS values represents an important limitation for clinical interpretation. Because GLS thresholds are increasingly used for diagnostic and prognostic decision-making, reliance on pooled reference ranges that do not account for sex differences may lead to misclassification of physiological versus pathological myocardial function. At the same time, understanding how deformation-derived measures relate to traditional indices such as LVEF is essential to contextualize their physiological meaning and clinical applicability. Establishing robust sex-specific

Sonaglioni et al. INPLASY protocol 202630007 doi:10.37766/inplasy2026.3.0007 Downloaded from https://inplasy.com/inplasy-2026-3-0007/

reference estimates is therefore essential to improve the precision of myocardial deformation assessment and support personalized cardiovascular evaluation.

Systematic synthesis of existing evidence is particularly warranted given the growing number of studies evaluating GLS in healthy individuals using both 2D-STE and 3D-STE methodologies across diverse populations. While individual investigations provide valuable normative data, variations in sample size and study design limit the generalizability of single-study findings. A meta-analytic approach allows integration of available evidence to derive weighted estimates that better reflect physiological myocardial deformation across populations and to compare deformation-based metrics with conventional systolic function indices within the same healthy cohorts.

Condition being studied Left ventricular global longitudinal strain (GLS), derived from speckle-tracking echocardiography (STE), has emerged as a sensitive and reproducible marker of myocardial systolic function, capable of detecting subtle alterations in myocardial mechanics beyond conventional parameters such as left ventricular ejection fraction (LVEF). While LVEF remains the cornerstone index of global systolic performance in clinical practice, deformation imaging provides complementary information by directly quantifying myocardial fiber shortening rather than volumetric chamber emptying. Over the last decade, GLS has progressively transitioned from a research tool to a clinically relevant parameter incorporated into routine echocardiographic assessment and international guideline recommendations for the evaluation of cardiomyopathies, cardio-oncology surveillance, and early myocardial dysfunction.

Despite its widespread adoption, defining normal reference values for GLS remains challenging. Numerous studies conducted in healthy populations using two-dimensional (2D) and three-dimensional (3D) STE have demonstrated considerable variability in reported GLS values. Differences in vendor technology, image acquisition protocols, post-processing algorithms, and population characteristics—including age, body size, and ethnicity—have contributed to heterogeneity across published reference ranges. Large population-based cohorts and multicenter investigations have confirmed that biological determinants significantly influence myocardial deformation measurements, highlighting the need for refined normative frameworks and for interpretation of deformation indices alongside established volumetric measures such as LVEF.

Among these determinants, biological sex has consistently emerged as one of the most relevant factors affecting myocardial mechanics. Several studies have reported higher absolute GLS values in women compared with men, even in the absence of structural heart disease, suggesting intrinsic sex-related differences in myocardial architecture, ventricular geometry, and myocardial fiber mechanics. Proposed mechanisms include smaller ventricular dimensions, differences in myocardial loading conditions, hormonal influences, and variations in myocardial contractile reserve. In contrast, sex-related differences in LVEF appear substantially smaller and less consistent across healthy populations, raising the possibility that myocardial deformation indices may capture physiological variation not fully reflected by conventional volumetric parameters. However, findings across studies remain partially inconsistent, with some cohorts reporting minimal or absent sex-related differences, likely reflecting methodological variability and population heterogeneity.

METHODS

Search strategy A comprehensive literature search was independently performed by two investigators to identify all studies reporting global longitudinal strain (GLS) values measured in healthy individuals using speckle-tracking echocardiography. Electronic databases including PubMed, Scopus, and EMBASE were systematically searched from database inception to February 2026. The search strategy combined controlled vocabulary terms and free-text keywords related to myocardial deformation imaging and normative populations. Search terms included combinations of “global longitudinal strain”, “GLS”, “myocardial strain”, “speckle tracking echocardiography”, “2D-STE”, “3D-STE”, “healthy subjects”, “normal population”, and “reference values”. No restrictions were applied regarding language, publication year, or geographic region.

Because the secondary objective of the study was to compare myocardial deformation with conventional systolic function indices, left ventricular ejection fraction (LVEF) data were additionally extracted from all eligible studies identified through the GLS-focused search strategy when such information was available. No separate search strategy was performed for LVEF, as the analysis was designed to evaluate complementary physiological information within the same healthy populations.

In addition, reference lists of all eligible articles and relevant review papers were manually screened to identify potentially relevant studies not captured through the electronic search. Discrepancies

Sonaglioni et al. INPLASY protocol 202630007 doi:10.37766/inplasy2026.3.0007 Downloaded from https://inplasy.com/inplasy-2026-3-0007/

between reviewers during the screening process were resolved by discussion and consensus, with consultation of a third investigator when required.

Participant or population Studies were considered eligible if they had an observational design, including cross-sectional or cohort studies, and evaluated myocardial deformation in healthy adult populations free of known cardiovascular disease. Eligible studies were required to assess GLS using echocardiographic techniques, specifically two-dimensional or three-dimensional speckle-tracking echocardiography (2D-STE or 3D-STE), and to provide extractable quantitative GLS data reported as mean ± standard deviation, median with dispersion measures, or in a format suitable for statistical transformation. Studies were additionally required to report sex-specific GLS values or provide sufficient information to allow comparison between women and men.

Studies reporting sex-stratified left ventricular ejection fraction (LVEF) values, or providing sufficient information to derive sex-specific LVEF comparisons, were additionally considered eligible for inclusion in the secondary quantitative synthesis aimed at comparing myocardial deformation with conventional systolic function indices.

Only studies evaluating left ventricular global longitudinal strain (LV-GLS) derived from echocardiographic speckle-tracking analysis in human subjects were included. Studies in which myocardial deformation was assessed using cardiac magnetic resonance (CMR)—including feature-tracking CMR, myocardial tagging, strain-encoded imaging (SENC), or other CMR-based strain techniques—were excluded because of fundamental differences in acquisition methodology, tracking algorithms, and modality-specific reference values compared with echocardiographic strain measurements.

Intervention N/A.

Comparator The primary objective of the quantitative synthesis was to evaluate sex-related differences in left ventricular global longitudinal strain (GLS) between healthy women and men. In addition, a secondary meta-analysis was performed to assess sex-related differences in left ventricular ejection fraction (LVEF), allowing comparison between myocardial deformation indices and conventional volumetric measures of systolic function.

Study designs to be included Observational Cohort and Cross-Sectional Studies.

Eligibility criteria Studies were considered eligible if they had an observational design, including cross-sectional or cohort studies, and evaluated myocardial deformation in healthy adult populations free of known cardiovascular disease. Eligible studies were required to assess GLS using echocardiographic techniques, specifically two-dimensional or three-dimensional speckle-tracking echocardiography (2D-STE or 3D-STE), and to provide extractable quantitative GLS data reported as mean ± standard deviation, median with dispersion measures, or in a format suitable for statistical transformation. Studies were additionally required to report sex-specific GLS values or provide sufficient information to allow comparison between women and men.

Studies reporting sex-stratified left ventricular ejection fraction (LVEF) values, or providing sufficient information to derive sex-specific LVEF comparisons, were additionally considered eligible for inclusion in the secondary quantitative synthesis aimed at comparing myocardial deformation with conventional systolic function indices.

Only studies evaluating left ventricular global longitudinal strain (LV-GLS) derived from echocardiographic speckle-tracking analysis in human subjects were included. Studies in which myocardial deformation was assessed using cardiac magnetic resonance (CMR)—including feature-tracking CMR, myocardial tagging, strain-encoded imaging (SENC), or other CMR-based strain techniques—were excluded because of fundamental differences in acquisition methodology, tracking algorithms, and modality-specific reference values compared with echocardiographic strain measurements.

For the complementary LVEF analysis, conventional echocardiographic measurements obtained using standard two-dimensional or three-dimensional imaging were considered eligible irrespective of strain vendor or tracking software, given the modality-independent nature of volumetric ejection fraction assessment.

Studies conducted in animal models or experimental preclinical settings were also excluded to ensure physiological comparability and clinical applicability of pooled reference values.

Furthermore, studies primarily focused on right ventricular global longitudinal strain (RV-GLS), left atrial strain parameters (including LASr), or deformation indices other than LV-GLS were not considered eligible.

Studies were excluded if they enrolled mixed populations without separable healthy cohorts, lacked quantitative GLS reporting, used experimental or non-standardized strain

Sonaglioni et al. INPLASY protocol 202630007 doi:10.37766/inplasy2026.3.0007 Downloaded from https://inplasy.com/inplasy-2026-3-0007/

methodologies, or provided insufficient data for pooled analysis. Non-original publications including editorials, conference abstracts, letters, case reports, narrative reviews, and guidelines were also excluded.
Studies lacking extractable quantitative LVEF data were excluded from the secondary LVEF meta-analysis but remained eligible for inclusion in the primary GLS synthesis.

Information sources A comprehensive literature search was independently performed by two investigators to identify all studies reporting global longitudinal strain (GLS) values measured in healthy individuals using speckle-tracking echocardiography. Electronic databases including PubMed, Scopus, and EMBASE were systematically searched from database inception to February 2026. The search strategy combined controlled vocabulary terms and free-text keywords related to myocardial deformation imaging and normative populations. Search terms included combinations of "global longitudinal strain", "GLS", "myocardial strain", "speckle tracking echocardiography", "2D-STE", "3D-STE", "healthy subjects", "normal population", and "reference values". No restrictions were applied regarding language, publication year, or geographic region.

Main outcome(s) The primary objective of the quantitative synthesis was to evaluate sex-related differences in left ventricular global longitudinal strain between healthy women and men.

Additional outcome(s) In addition, a secondary meta-analysis was performed to assess sex-related differences in left ventricular ejection fraction, allowing comparison between myocardial deformation indices and conventional volumetric measures of systolic function.

Quality assessment / Risk of bias analysis Methodological quality and risk of bias of included studies were independently evaluated by two reviewers using the National Institutes of Health (NIH) Quality Assessment Tool for Observational Cohort and Cross-Sectional Studies. This tool examines key methodological domains including study population definition, eligibility criteria, exposure and outcome assessment, consistency of measurement methods, statistical analysis, and completeness of reporting.
Each study was assessed across 14 domains and rated as "Yes," "No," "Cannot determine," "Not reported," or "Not applicable," following NIH guidance. Affirmative responses were considered indicative of fulfilled quality criteria, while items deemed not applicable were excluded from score calculation. A summary score was obtained by counting the number of criteria satisfied.
Overall methodological quality was classified according to predefined thresholds: studies fulfilling 9–14 criteria were rated as good quality, those meeting 5–8 criteria as fair quality, and those fulfilling fewer than 5 criteria as poor quality. Final classification incorporated both the numerical score and reviewer judgment regarding key methodological domains.
Inter-rater agreement between reviewers was quantified using Cohen's kappa coefficient. Any disagreement in domain ratings or overall quality classification was resolved through discussion and re-evaluation of the original manuscripts until consensus was reached. Methodological quality results were summarized using graphical risk-of-bias representations.

Strategy of data synthesis Two investigators independently screened all retrieved records by title and abstract, followed by full-text assessment of potentially eligible studies according to predefined inclusion and exclusion criteria. Disagreements regarding eligibility were resolved through discussion and consensus, and when necessary a third reviewer adjudicated disagreements.
Data extraction was independently performed using a standardized data collection form developed a priori. Extracted variables included study characteristics (first author, publication year, country, study design, imaging modality, and sample size).
Demographic and anthropometric parameters included age, sex distribution, body mass index, body surface area, and cardiovascular risk profile variables when available (including blood pressure values, heart rate, smoking prevalence, serum cholesterol, and glucose levels).
Clinical and conventional echocardiographic variables were systematically collected to characterize cardiac structure and function in healthy populations. These included interventricular septal thickness, posterior wall thickness, left ventricular end-diastolic diameter, relative wall thickness, left ventricular mass index, left ventricular end-diastolic and end-systolic volumes, left ventricular ejection fraction, left atrial volume index, and right ventricular structural and functional parameters such as basal right ventricular diameter, tricuspid annular plane systolic excursion (TAPSE), and estimated pulmonary pressure indices when reported.
Sex-specific LVEF values for women and men were extracted whenever available. When studies reported only overall LVEF values or reference

Sonaglioni et al. INPLASY protocol 202630007 doi:10.37766/inplasy2026.3.0007 Downloaded from https://inplasy.com/inplasy-2026-3-0007/

limits, additional information allowing derivation or reconstruction of sex-specific summary statistics was recorded for subsequent quantitative synthesis.
Diastolic function parameters were also extracted, including transmitral E/A ratio and E/e′ ratio.
Strain-derived measures included global longitudinal strain (GLS) values, sex-specific GLS estimates for women and men, and additional deformation parameters when available, including global circumferential strain, global radial strain, right ventricular longitudinal strain indices, and view-specific longitudinal strain measurements derived from apical four-chamber, two-chamber, and three-chamber views. Segmental longitudinal strain values were additionally recorded when reported, including basal, mid-ventricular, and apical strain measurements.
When numerical values were available only in graphical format, data were extracted using digital plot analysis software. All extracted data were cross-checked for accuracy by both reviewers, and discrepancies were resolved through re-evaluation of original manuscripts until agreement was achieved.

Subgroup analysis Pre-specified subgroup analyses were conducted according to imaging modality. For GLS analyses, studies were stratified according to speckle-tracking dimensionality (two-dimensional speckle-tracking echocardiography [2D-STE] versus three-dimensional speckle-tracking echocardiography [3D-STE]). For LVEF analyses, subgroup comparisons were performed according to echocardiographic acquisition technique (two-dimensional versus three-dimensional echocardiography). Separate random-effects models were constructed for each subgroup, and between-subgroup differences were evaluated using the Q-test for subgroup interaction.

Sensitivity analysis Sensitivity analyses were performed using a leave-one-out approach to evaluate the robustness of pooled estimates for both GLS and LVEF analyses.

Language restriction No language restriction.

Country(ies) involved Italy.

Keywords global longitudinal strain; speckle-tracking echocardiography; sex differences; myocardial deformation; reference values; meta-analysis.

Contributions of each author

Author 1 - Andrea Sonaglioni - Author 1 drafted the manuscript.
Email: sonaglioniandrea@gmail.com
Author 2 - Giulio Francesco Gramaglia - The author contributed to the search strategy.
Email: giulio.gramaglia@unimi.it
Author 3 - Gian Luigi Nicolosi - The author revised the first version of the manuscript.
Email: gianluigi.nicolosi@gmail.com
Author 4 - Massimo Baravelli - The author read, provided feedback and approved the final manuscript.
Email: massimo.baravelli@multimedica.it
Author 5 - Michele Lombardo - Supervision.
Email: michele.lombardo@multimedica.it

Sonaglioni et al. INPLASY protocol 202630007 doi:10.37766/inplasy2026.3.0007 Downloaded from https://inplasy.com/inplasy-2026-3-0007/
