# Supplementary material for: Sex-Related Differences in Myocardial Deformation and Systolic Function in Healthy Individuals: A Systematic Review and Meta-Analysis of Global Longitudinal Strain and Left Ventricular Ejection Fraction
Source: J Clin Med. 2026 Apr 9;15(8):2859. doi: 10.3390/jcm15082859 (PMC13115635; doi:10.3390/jcm15082859)
Supplement: Supplementary file 1 [file jcm-15-02859-s001.zip › Supplementary Materials S3.pdf]

**Study name**	**Q1**	**Q2**	**Q3**	**Q4**	**Q5**	**Q6**	**Q7**	**Q8**	**Q9**	**Q10**	**Q11**	**Q12**	**Q13**	**Q14**	**Yes**	**Overall**
Saito 2009 [22]	Y	Y	NR	Y	N	NA	NA	NA	Y	N	Y	NR	NA	NA	5	Fair
Dalen 2010 [23]	Y	Y	Y	Y	Y	NA	NA	Y	Y	N	Y	NR	NA	Y	9	Good
Takigiku 2012 [24]	Y	Y	NR	Y	NR	NA	NA	Y	Y	N	Y	NR	NA	Y	7	Good
Kaku 2014 [25]	Y	Y	NR	Y	NR	NA	NA	Y	Y	N	Y	NR	NA	Y	7	Good
Kocabay 2014 [26]	Y	Y	NR	Y	NR	NA	NA	Y	Y	N	Y	NR	NA	Y	7	Good
Muraru 2014 [27]	Y	Y	NR	Y	Y	NA	NA	Y	Y	N	Y	NR	NA	Y	8	Good
Xu 2014 [28]	Y	Y	NR	N	NR	NA	NA	Y	Y	N	Y	NR	NA	N	5	Fair
Kleijn 2015 [29]	Y	Y	NR	Y	Y	NA	NA	Y	Y	N	Y	NR	NA	Y	8	Good
Shi 2016 [30]	Y	Y	NR	Y	NR	NA	NA	Y	Y	N	Y	NR	NA	Y	7	Good
Park 2016 [31]	Y	Y	NR	Y	Y	NA	NA	Y	Y	N	Y	NR	NA	Y	8	Good
Bernard 2017 [32]	Y	Y	Y	Y	Y	NA	NA	Y	Y	N	Y	NR	NA	Y	9	Good
Nagata 2017 [33]	Y	Y	NR	Y	NR	NA	NA	Y	Y	N	Y	NR	NA	Y	7	Good
Støylen 2018 [34]	Y	Y	Y	Y	Y	NA	NA	Y	Y	N	Y	NR	NA	Y	9	Good
Sullere 2018 [35]	Y	Y	NR	Y	NR	NA	NA	Y	Y	N	Y	NR	NA	Y	7	Good
Alcidi 2017 [36]	Y	Y	NR	Y	NR	NA	NA	Y	Y	N	Y	NR	NA	Y	7	Good
Saraiva 2019 [37]	Y	Y	NR	Y	N	NA	NA	Y	Y	N	Y	NR	NA	Y	6	Fair
Tsugu 2020 [38]	Y	Y	NR	Y	Y	NA	NA	Y	Y	N	Y	NR	NA	Y	8	Good
Faganello 2020 [39]	Y	Y	NR	Y	NR	NA	NA	Y	Y	N	Y	NR	NA	Y	7	Good
Potter 2021 [40]	Y	Y	NR	N	NR	NA	NA	N	Y	N	Y	NR	NA	N	4	Poor
Sengupta 2021 [41]	Y	Y	NR	Y	Y	NA	NA	Y	Y	N	Y	NR	NA	Y	8	Good
Nemes 2021 [42]	Y	Y	NR	Y	NR	NA	NA	Y	Y	N	Y	NR	NA	Y	7	Good
Ferrara 2021 [43]	Y	Y	NR	Y	NR	NA	NA	Y	Y	N	Y	NR	NA	Y	7	Good
Wegener 2022 [44]	Y	Y	NR	Y	NR	NA	NA	Y	Y	N	Y	NR	NA	Y	7	Good
Skaarup 2022 [45]	Y	Y	Y	Y	Y	NA	NA	Y	Y	N	Y	NR	NA	Y	9	Good
Addetia 2022 [46]	Y	Y	NR	Y	Y	NA	NA	Y	Y	N	Y	NR	NA	Y	8	Good
Morais 2022 [47]	Y	Y	NR	Y	NR	NA	NA	Y	Y	N	Y	NR	NA	Y	7	Good
Kornev 2022 [48]	Y	Y	Y	Y	Y	NA	NA	Y	Y	N	Y	NR	NA	Y	9	Good
Nyberg 2023 [49]	Y	Y	Y	Y	Y	NA	NA	Y	Y	N	Y	NR	NA	Y	9	Good
Moraru 2024 [50]	Y	Y	NR	Y	NR	NA	NA	Y	Y	N	Y	NR	NA	Y	7	Good
Wang 2024 [51]	Y	Y	NR	Y	Y	NA	NA	Y	Y	N	Y	Y	NA	Y	9	Good
Arockiam 2025 [52]	Y	Y	NR	Y	NR	NA	NA	Y	Y	N	Y	NR	NA	Y	7	Good
Kotini-Shah 2026 [53]	Y	Y	Y	Y	Y	NA	NA	Y	Y	N	Y	NR	NA	Y	9	Good
