# Supplementary figures and images for: Sex-Related Differences in Myocardial Deformation and Systolic Function in Healthy Individuals: A Systematic Review and Meta-Analysis of Global Longitudinal Strain and Left Ventricular Ejection Fraction
Source: J Clin Med. 2026 Apr 9;15(8):2859. doi: 10.3390/jcm15082859 (PMC13115635; doi:10.3390/jcm15082859)

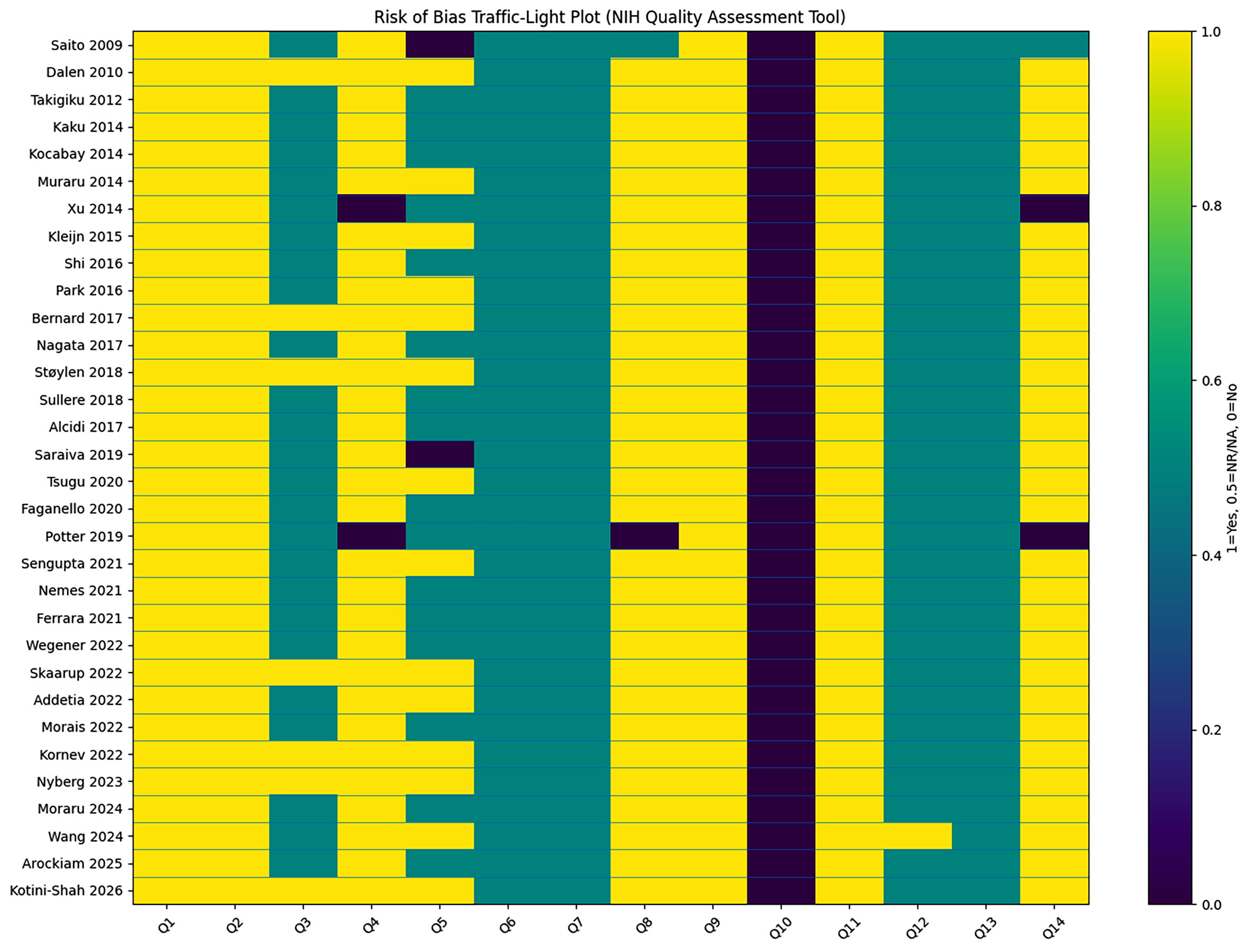

Supplement: Supplementary file 1 [file jcm-15-02859-s001.zip › Supplementary Materials S4.png]

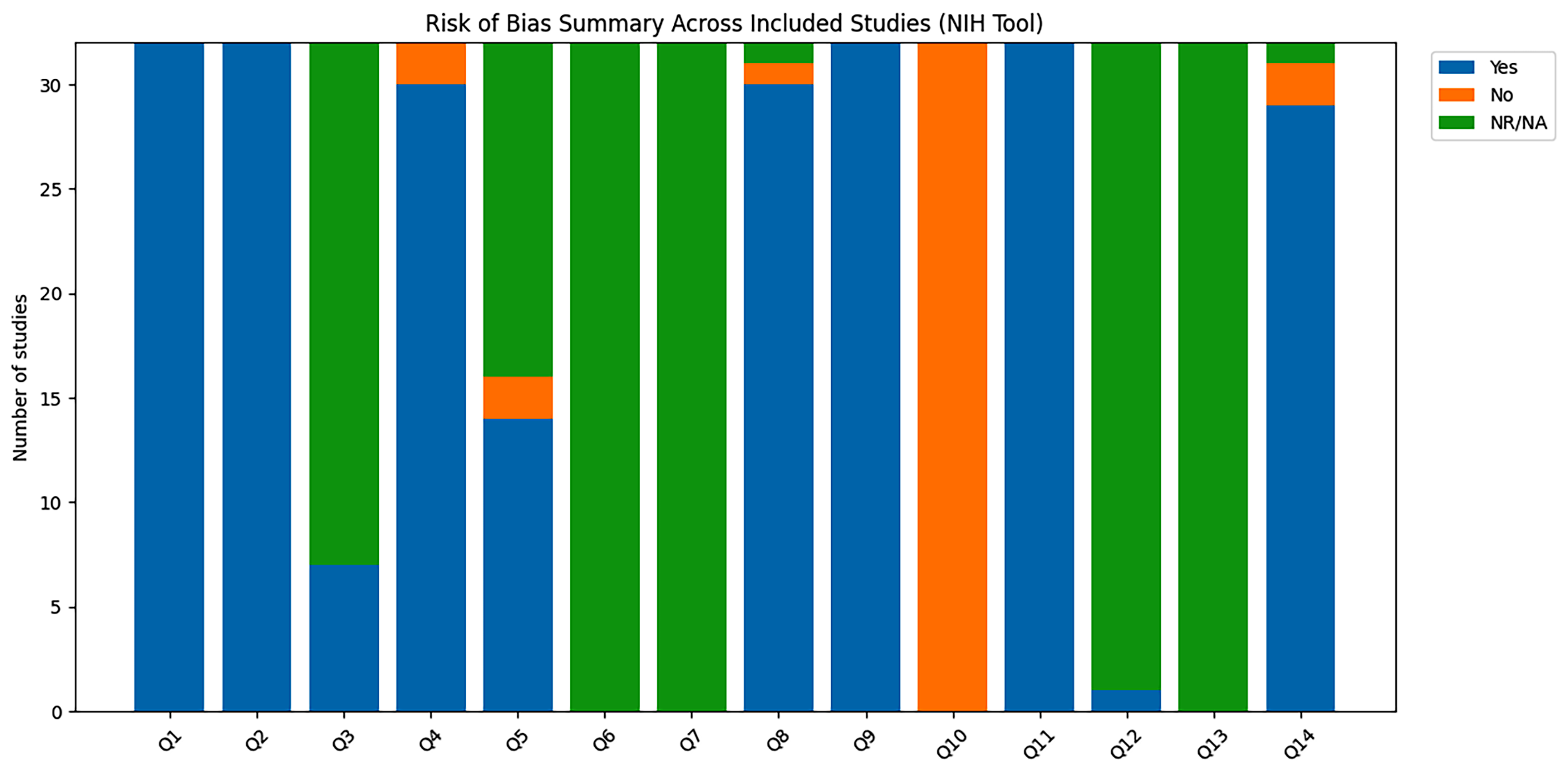

Supplement: Supplementary file 1 [file jcm-15-02859-s001.zip › Supplementary Materials S5.png]
